# Peas, natural resources for a sustainable future: a multifaceted review of nutritional, health, environmental, and market perspectives

**DOI:** 10.3389/fnut.2025.1703760

**Published:** 2026-01-12

**Authors:** Nada Ćujić Nikolić, Zorana Mutavski, Katarina Šavikin, Jelena Živković, Suzana Pavlović, Petra Jones, Claire Copperstone, Erdi Can Aytar, Betül Aydin, Evelien Van Bavegem, Ibrahim Ender Kunili, Özge Özmen, Aylin Seylam Küşümler, Derya Ozalp Unal, Selin Gunduz, Szymon Wojciech Lara, Meleksen Akin, Amil Orahovac, Bálint Balázs, Jelena Milešević, Alexandrina Sîrbu, Sonia Negrão, Marija Knez

**Affiliations:** 1 Department for Pharmaceutical Research and Development, Institute for Medicinal Plants Research “Dr Josif Pančić”, Belgrade, Serbia; 2 Center of Research Excellence in Nutrition and Metabolism, Institute for Medical Research, National Institute of the Republic of Serbia, University of Belgrade, Belgrade, Serbia; 3 Department of Food Science, Nutrition and Dietetics, Faculty of Health Sciences, University of Malta, Msida, Malta; 4 Usak University, Faculty of Agriculture, Department of Horticulture, Uşak, Türkiye; 5 Gazi University, Faculty of Science, Department of Biology, Ankara, Türkiye; 6 Research Centre AgroFoodNature, HOGENT University of Applied Sciences and Arts, Ghent, Belgium; 7 Faculty of Marine Science and Technology, Department of Fishing and Processing Technology, Çanakkale Onsekiz Mart University, Çanakkale, Turkey; 8 Department of Food Engineering, Faculty of Engineering, Adana Alparslan Türkeş Science and Technology University, Adana, Türkiye; 9 Istanbul Okan University, Faculty of Health Sciences, Nutrition and Dietetics Department, Istanbul, Türkiye; 10 Institute of Field Crops Central Research, Ministry of Agriculture and Forestry, Republic of Türkiye, Ankara, Türkiye; 11 Royal Botanic Gardens, Kew, Richmond, London, United Kingdom; 12 London Geller College of Hospitality and Tourism, University of West London, London, United Kingdom; 13 Horticulture Department, Faculty of Agriculture, Igdir University, Igdir, Türkiye; 14 Faculty of Food Safety, Food Technology and Ecology, University of Donja Gorica, Podgorica, Montenegro; 15 ESSRG Nonprofit Ltd, Budapest, Hungary; 16 FMMAE Râmnicu Vâlcea, Constantin Brâncoveanu University of Piteşti, Râmnicu Vâlcea, Romania; 17 School of Biology and Environmental Science, University College Dublin, Dublin, Ireland

**Keywords:** *Pisum sativum* L., peas, sustainable food systems, nutritional composition, bioactive compounds, health-promoting properties, legume sustainability, antinutritional

## Abstract

The pea (*Pisum sativum* L.) is an emerging pillar in plant-based nutrition and sustainable food systems due to its high-quality proteins, diverse bioactive compounds, and agroecological benefits. This review provides an updated synthesis of the nutritional composition, health-promoting properties, and environmental relevance of peas, emphasizing recent scientific findings. Pea seeds typically contain 20%−40% protein, 45%−55% starch, and 10%−15% dietary fiber, alongside essential micronutrients such as vitamin C (40–60 mg/100 g), folate (60–70 μg/100 g), vitamin K (30–45 μg/100 g), iron (1.5–2.0 mg/100 g), and manganese (0.4–0.6 mg/100 g). Their storage proteins, primarily legumin and vicilin, offer high digestibility and amino acid profiles compatible with human requirements, supporting their rapidly growing use in protein isolates and meat- and dairy-alternative products. Peas represent a valuable source of phenolic acids, flavonoids, and saponins, which contribute to notable antioxidant (50–120 μmol Trolox/g) and anti-inflammatory activities demonstrated in preclinical studies. Compared with other legumes, peas exhibit a lower glycemic index (35–45), making them suitable for metabolic health applications. Agronomically, pea cultivation enhances soil fertility through biological nitrogen fixation (up to 150 kg N/ha), supporting reduced fertilizer inputs and improved crop rotation performance, aligning with circular economy and climate-resilience strategies. Despite these advantages, global consumption and breeding innovation remain insufficient to meet the rising demand for alternative proteins. Future opportunities include improving protein extraction technologies, valorizing processing side-streams, and exploring underutilized phytochemicals to strengthen the nutritional and sustainability profile of pea-based food systems.

## 1 Introduction

Pea (well-known as *Pisum sativum* L.), currently reclassified taxonomically as *Lathyrus oleraceus* Lam. syn *P. sativum* L., is one of the most widely cultivated legumes in the world and has long played an essential role in the human diet. Its popularity is due not only to its agronomic adaptability and favorable cultivation characteristics, but also to its rich nutritional composition and broad applicability in food systems (1). Although botanically a legume, the pea is primarily consumed in its immature form as a vegetable, valued for its mild sweetness, delicate texture, and bright color, and continues to attract scientific and industrial interest due to its nutritional and functional potential.

Growing global interest in plant-based diets has increased the demand for peas as a sustainable source of plant proteins, complex carbohydrates, and dietary fiber (2), offering many health benefits. Peas are also particularly rich in micronutrients, including vitamin C, vitamin K, folate, copper, manganese, iron, zinc, phosphorus, and magnesium (3). Beyond their macronutrient and micronutrient composition, peas are rich in bioactive compounds such as phenolic acids, flavonoids, and saponins that exhibit antioxidant, anti-inflammatory, and potential anti-cancer effects, supporting vital physiological processes such as immune function and cellular activity (4), making peas a functional food with disease-preventive potential. The presence of these bioactives enhances their role not only in basic nutrition but also in strategies aimed at reducing the burden of chronic, non-communicable diseases (5).

The macro and micronutrient composition, specifically bioactives, have been associated with improved glycemic regulation, enhanced gastrointestinal function, support hematological, neurological, and skeletal health, and protection against oxidative and metabolic stress (1, 5–7). Scientific research, including both *in vitro* and *in vivo* studies, has demonstrated that these compounds may help reduce the risk of cardiovascular disease, obesity, and certain types of cancer. Notably, peas have a low glycemic index, making them suitable for people who need to manage their blood sugar levels, including those with insulin resistance or type 2 diabetes (8). Moreover, underutilized components such as pea pods and seed coats are emerging as valuable sources of fiber and phenolic compounds with demonstrated antidiabetic, hepatoprotective, and renoprotective activities (9). Emerging evidence further indicates that pea-derived peptides and dietary fibers may modulate the gut microbiome, supporting microbial diversity and metabolic balance, thereby contributing to their overall health-promoting effects (10). These characteristics highlight their potential role in the development of functional foods, sustainable nutrition strategies, and improving global food and nutrition security.

Beyond their nutritional and health benefits, peas have also gained significant attention for their ecological and economic value, especially in the context of sustainable and circular food systems. Environmentally, peas play a key role in improving soil health due to their nitrogen-fixing properties. As a legume, they absorb nitrogen from the atmosphere, enriching the soil and reducing the reliance on synthetic fertilizers, which supports more sustainable farming practices (11–13). In addition, the growing focus on circular economy principles highlights the need to maximize resource efficiency and minimize waste across the food chain (14). Pea production and processing generate nutrient-rich by-products, such as pods and husks, that are often discarded despite their nutritional potential (9, 15). Valorizing these materials as food ingredients or bio-based materials aligns with circular food system goals, reducing waste while enhancing economic and environmental sustainability (16, 17). These environmental advantages complement their emerging use in modern food innovation through upcycling, where peas are utilized in applications ranging from minimally processed meals to highly formulated plant-based products.

**Figure 1:**
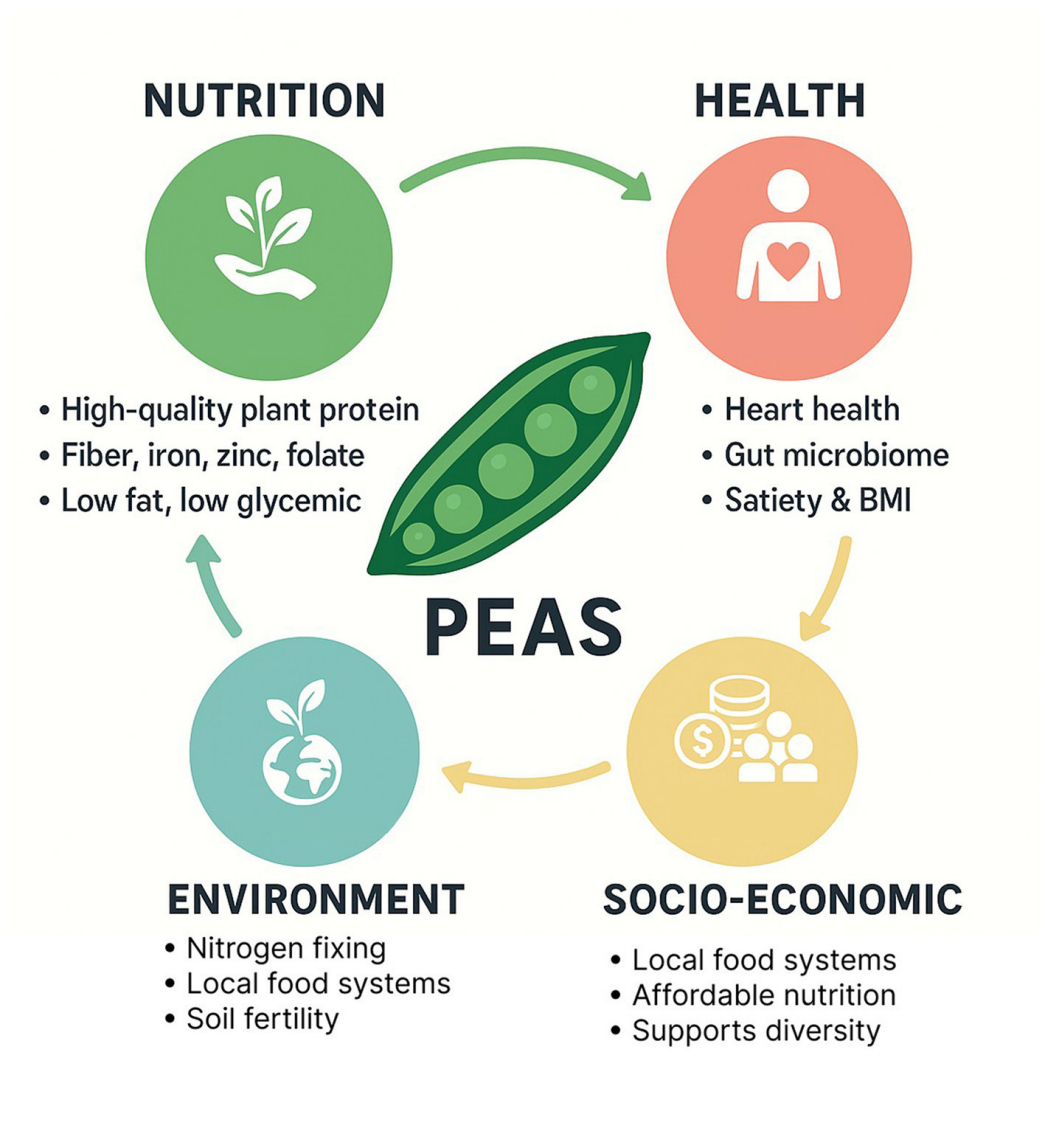
Insights of different pea range of applications.

With the rising demand for sustainable, nutritious, and versatile ingredients, peas are used in a wide range of food applications, including plant-based beverages, meat analogs, and flour-based products (18–20), and innovative delivery systems like encapsulated bioactives and biodegradable packaging (21). These applications underscore their versatility and technological potential in modern food innovation, positioning pea as one of the key ingredients for the future of food production. These technological advances not only enhance the functional versatility of peas but also have broader implications for nutrition security and public health. Beyond individual health benefits, the properties of peas also impact public health strategies and the development of functional foods (22). As an affordable, accessible, and nutrient-dense food, peas can help address global issues like food insecurity (20), micronutrient deficiencies and diet-related chronic diseases. Their environmental compatibility and potential role in circular food systems position peas as a cornerstone of sustainable and health-promoting diets (Figure 1).

With growing global interest in sustainable, health-conscious diets, a comprehensive understanding of the nutritional and therapeutic potential of peas is more crucial than ever. Despite these diverse benefits and applications, comprehensive insights linking the compositional traits, health effects, and sustainability potential of peas remain limited. Existing literature often addresses these aspects in isolation, leaving a gap in understanding how peas can simultaneously contribute to nutrition security, environmental resilience, and circular bioeconomy goals. To address these gaps, this review synthesizes current evidence on the nutritional composition, bioactive compounds, physiochemical properties, and health benefits of peas; examines their technological and functional applications in sustainable food innovation; and highlights opportunities for valorizing underutilized pea components within circular economy frameworks. Given its growing importance as a sustainable, nutrient-rich, and multifunctional legume, this article examines the full range of peas' value, from their fundamental nutritional contributions to their emerging roles in disease prevention and the treatment of various health conditions. This review aims to position peas as a strategic crop at the intersection of human and planetary health, as well as environmental sustainability. Additionally, the insights gained from this review could inform and facilitate policy development, supporting the creation of frameworks that promote sustainable agricultural practices, enhance food security, and drive the transition to circular food systems. By integrating perspectives from nutrition, agronomy, food technology, and public health, this review aims to position peas as a strategic crop at the intersection of human and planetary health, as well as environmental sustainability. Additionally, the insights gained from this review could inform and facilitate policy development, supporting the creation of frameworks that promote sustainable agricultural practices, enhance food security, and drive the transition to circular food systems (Tables 1, 2).

**Table 1:** Historical and modern utilization of peas and their significance.

**Period**	**Timeline**	**Utilization**	**Significance**
Ancient period	≈5000–1000 BCE	Domestication and early cultivation; staple food; dried peas stored for winter.	Primary protein source; important in early agriculture; early recognition of storage and food security.
Classical & medieval period	1,000 BCE−1,500 CE	Common in soups and porridges; crop rotation for soil fertility; medicinal uses.	Early agronomic knowledge; peas as functional food; contribution to soil nitrogen.
Early modern period	1500-1800 CE	Selective breeding (Mendel's experiments 1850s−1860s); fresh and dried peas in European cuisine.	Discovery of inheritance patterns; diversification in culinary use.
Industrial era	1800-1950	Canning and preservation; livestock feed; recognition of nutritional value.	Extension of shelf-life; protein source for humans and animals; industrial food applications.
Modern period	1950-Present	Food industry: pea protein isolates, snacks, soups, plant-based dairy alternatives; health/functional foods: bioactive compounds, antioxidants; sustainable agriculture: nitrogen-fixing crop, crop rotation; circular economy integration; biotechnological breeding: high-protein, low-antinutrient, climate-resilient varieties.	Functional and nutraceutical applications; contribution to sustainable food systems; bioactive compound research; climate-resilient agriculture; supports plant-based diets.

**Table 2:** Overview of global pea production, nutritional value, and commercialization challenges.

**Aspect**	**Details**
Global pea production	~10.5 million tons (dry peas), ~7 million tons (fresh peas)
Regional significance	Most commonly produced pulse in Europe and Central Asia
Nutritional value	High in plant-based proteins, fiber, and essential nutrients
Climate requirements	Prefers cool climates; sensitive to soil salinity
Agricultural challenges	Crop rotation needed; yield affected by weather conditions
Market challenges	Price volatility, limited consumer awareness, and underutilization
Trends in plant-based foods	Growing consumer interest, increasing availability of plant-based products, and product innovation in meat/dairy alternatives

**References:** FAO (217–219).

## 2 Materials and methods

The literature search was conducted electronically using the Scopus database in June 2024. Scopus was selected as the primary database for this review as it offers a comprehensive and multidisciplinary coverage of peer-reviewed journal articles across relevant disciplines. In line with the established guidance on systematic review methodology (23) Scopus also provides a robust search functionality, including advanced evidence search options.

### 2.1 Boolean code

The literature search was conducted using the following Boolean code: “Pisum sativum” AND (“functional food^*^” OR nutraceutic^*^ OR vitamin^*^ OR antioxidant^*^ OR polyphenol^*^ OR phenol^*^ OR flavonoid^*^ OR flavan^*^ OR anthocyanin^*^ OR metabolomic^*^ OR phytochemical^*^ OR “organic ac-id^*^” OR “secondary metabolites” OR bioactivity OF “bioactive compound^*^” OR Carbohydrate^*^ OR fiber^*^ OR protein^*^ OR “amino acid^*^” OR “fatty acid” OR mineral^*^ OR macroelement^*^ OR microelement^*^ OR macronutrient^*^ OR micronutrient^*^ OR vitamin^*^ OR ^*^toxin^*^ OR anti-nutrient^*^ OR antinutrient^*^ OR “protease inhibitor^*^” OR phytate^*^ OR “phytic acid” OR oxalate^*^ OR lectin^*^ OR tannin^*^ OR saponin^*^ OR amylase^*^ OR oligosaccharide^*^ OR trypsin^*^) AND (health OR pharmacol^*^ OR diet^*^ OR nutrition^*^) AND NOT (“grass pea^*^” OR “butterfly pea” OR “zombi pea” OR fish^*^ OR aquaculture).

The searches were limited to peer-reviewed and published journal articles only; gray literature was not considered for inclusion. The searches were not limited by any date restraints, geographical location, or area of study. Articles were, although required to be written in English.

### 2.2 Shortlisting, screening, and selection of evidence

Following the execution of the Boolean search, we conducted an additional shortlisting step, an in-text keyword verification using the same keywords as the original search string, including PubMed and Web of Science. We screened records to remove items that have been retrieved through indexing or algorithmic noise but did not actually contain the specified terms in the title, abstract, or full text.

All retrieved records underwent a two-stage screening process. Stage 1 comprised title and abstract screening to assess apparent relevance. Records judged potentially relevant were advanced to stage 2 screening, in which the full text was assessed against the eligibility criteria. Data from shortlisted studies were extracted into Excel 2016 for full-text screening and synthesis. At each screening stage, two reviewers independently assess records, with any disagreement resolved by a third reviewer.

### 2.3 Inclusion and exclusion criteria

Inclusion criteria were used throughout the search, shortlisting, and selection of relevant evidence. Studies were eligible for inclusion if they contained primary or secondary evidence that reported or mentioned the nutritional, bioactive, and physicochemical composition of peas (*Pisum sativum* L.).

On the other hand, studies were excluded if they (i) mentioned peas only incidentally without further analysis of their composition; (ii) focused on non-composition aspects of peas (e.g., agronomic practices, cultivation, processing technologies) without a direct link to the specified composition themes, or (iii) investigated plant species other than *Pisum sativum* L.

### 2.4 Data presentation

Data from all eligible studies were synthesized descriptively and organized into thematic sections across the manuscript: (i) the composition and nutritional profile of peas, (ii) health benefits associated with pea consumption, (iii) toxins and antinutritional factors in peas, and (iv) comparisons with other legumes.

### 2.5 PRISMA flow diagram

**Figure 2:**
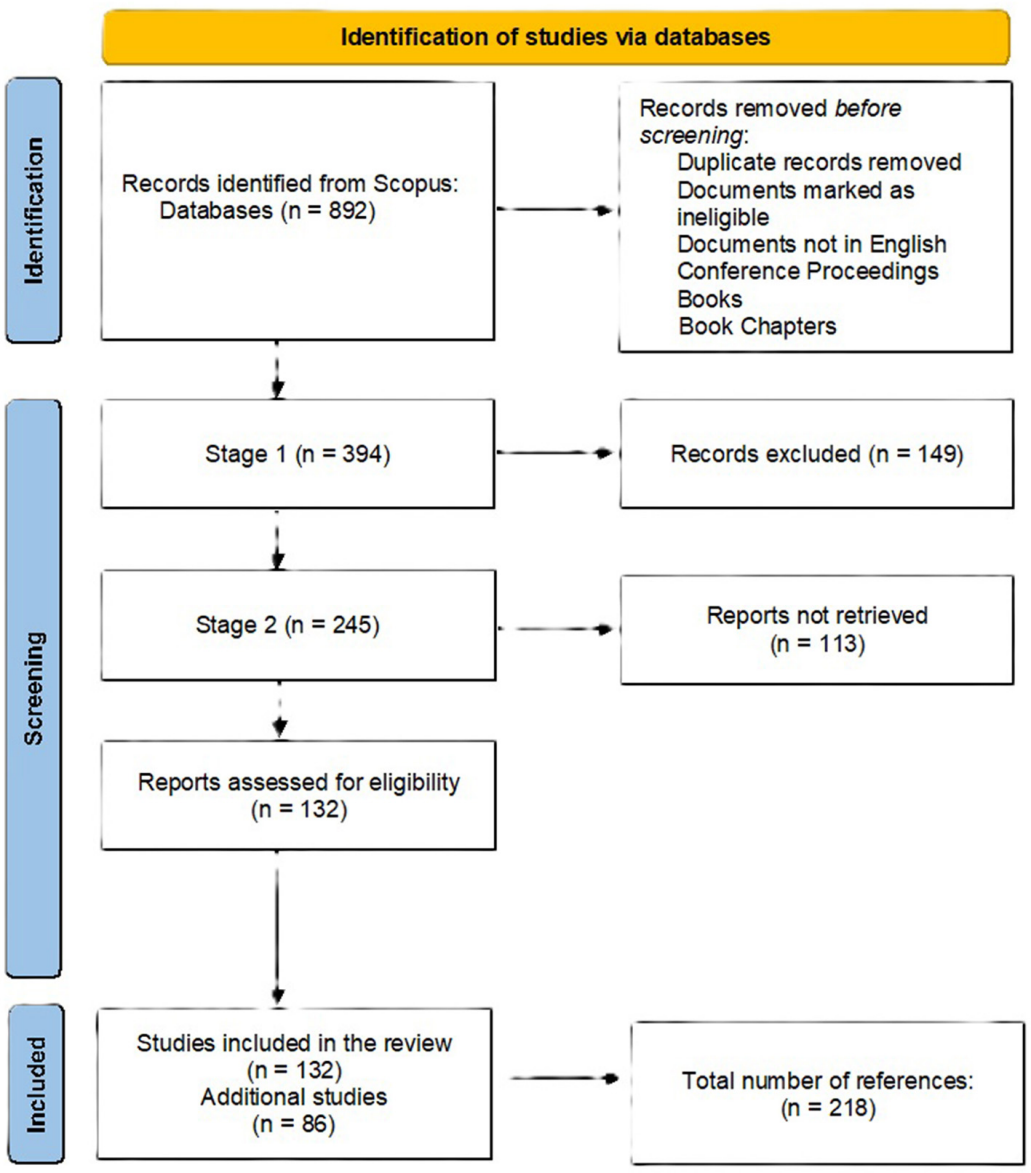
PRISMA flow diagram.

The literature searching strategy is presented using the PRISMA diagram. This review is structured as a narrative synthesis of the literature, incorporating certain systematic elements such as database search and predefined inclusion criteria. However, it does not adhere to the full PRISMA guidelines, which require a registered protocol, a comprehensive search strategy, risk of assessment, and a standardized flow diagram. Therefore, the present work should be considered a narrative review with structured components, rather than a systematic review. The flow diagram included is inspired by the PRISMA framework and is intended to provide transparency in the selection process, but it does not imply full compliance with PRISMA standards (Figure 2).

### 2.6 Additional references

Literature strategy was performed during June 2024, and manuscript preparation was performed from August 2024 to January 2025, with the available literature so far. Additional research included relevant references published in 2025.

### 2.7 Ethical considerations

Ethical approval was not required for this review.

## 3 Pea composition and nutritional profile

Whole peas are nutrient-dense legumes with a well-balanced profile of macronutrients, micronutrients, and bioactive compounds. As a source of high-quality plant protein, peas provide essential amino acids, particularly lysine and arginine, which are often limited in cereal-based diets (24). While peas have a high lysine content, they lack amino acids with thiol groups. In addition to protein, peas are rich in dietary fiber, including both soluble and insoluble fractions (such as starches and non-starch polysaccharides), which can contribute to digestive health and metabolic regulation. Among many bioactive phytochemicals, peas contain a variety of polyphenols, such as phenolic acids, flavonoids, and saponins, all of which have well-documented antioxidant and anti-inflammatory effects. The differences in the colors of the pea coat are usually associated with flavonoid content, and dark-seed coat samples generally have a higher flavonoid content than light-colored samples. The peas' exact composition can vary depending on the cultivar, maturity stage, and processing method, but overall, peas are recognized as a multifunctional food ingredient with both nutritional and functional value (5). Besides the mentioned compounds, peas also contain anti-nutritional factors, such as phytic acid, lectin, and trypsin inhibitors, which may interfere with nutrient bioavailability (Table 3).

**Table 3:** Nutritional and phytochemical constituents of peas.

**Compound class**	**Specific compounds/examples**	**Notes**	**References**
Carbohydrates	Starch (amylose, amylopectin), total sugars, resistant starch	Starch content: 39%−69% DW; RS up to 5–53 g/kg	(25, 26)
Proteins	Globulins (legumin, vicilin), albumins, glutelins, prolamins	13%−38% DW; high digestibility; legumin/vicilin ~2:1	(28, 37)
Lipids	Linoleic acid, α-linolenic acid, oleic acid, palmitic acid	1.4–1.9 g/100 g DW; rich in PUFA	(26, 43, 116)
Dietary Fiber	Cellulose, hemicellulose, lignin	Present in moderate quantities	(26, 220)
Minerals	Fe, Zn, Mg, Ca, K, P	Contribute to nutritional and functional properties	(57, 129)
Vitamins	C, E, B-complex (especially folate), K	Mostly in fresh green peas	(55)
Phenolic Acids	Protocatechuic, vanillic, ferulic, p-coumaric, gentisic, syringic, caffeoylquinic acids, rosmarinic acid	Higher concentration in seed coats, especially in dark-colored varieties	(50, 64, 65)
Flavonoids	Epigallocatechin, luteolin glycoside, apigenin glycoside, quercetin-3-O-rhamnoside, morin, naringin, chrysin, rutin, pinocembrin	Higher in dark-colored seed coats; contributes to antioxidant capacity	(55, 63, 65)
Other Polyphenols	Galangin, hesperetin, tetrahydroxy dihydrochalcone	Detected in various seed coat extracts	(50, 65)
Carotenoids	All-trans-lutein, all-trans-zeaxanthin, 13-cis-lutein, 15-cis-lutein	TCC up to 29.61 ± 4.46 mg/g (yellow split pea)	(43)
Amino Acids	Lysine, arginine, leucine, isoleucine, glutamic acid, and aspartic acid	Pea proteins are rich in lysine, poor in sulfur amino acids	(28)
Tannins	Catechin-type, condensed tannins	In the seed coat, influences digestibility and color	(55, 61)

### 3.1 Macronutrient content (proteins, carbohydrates, fats)

Among macronutrients in peas, carbohydrates primarily consist of slowly digestible starches and resistant starch, which support glycemic control. The fat content of peas is naturally low and consists mainly of unsaturated fatty acids. Peas are among the richest protein-rich legumes, comparable to lentils and chickpeas. When paired with cereal proteins (e.g., wheat or rice), pea protein can help create a complete amino acid profile in plant-based diets.

#### 3.1.1 Carbohydrates

Carbohydrates are among the primary chemical constituents of peas, comprising approximately 59.32%−69.59% of the dry seed weight. Starch is a significant carbohydrate fraction, predominantly composed of amylose and amylopectin, whose ratio markedly affects its physicochemical properties. Pea starch is characterized by a relatively high amylose content, ranging from 17.2% to 42.6%, with wrinkled peas generally containing more amylose than round ones (4).

Santos et al. (25) highlighted the high resistant starch content in yellow peas, noting its potential benefits for gut health and its contribution to a low-glycemic dietary profile.

Cervenski et al. (26) emphasized the importance of selecting parents appropriately in breeding programs aimed at enhancing the chemical composition of pea seeds. Their study examined technologically mature seeds from a winter pea collection and assessed various traits, including protein, total nitrogen, total sugars, starch, crude fat, cellulose, and ash content. In this collection, the starch content ranged from 39.44 to 46.23 g/100 g. For comparison, the average starch content in extruded gray pea products was reported as 23 ± 2 g/100 g (27).

#### 3.1.2 Proteins

Proteins are one of the main constituents of pea seeds. Its levels in pea seed range from 20% to 30% (28), but there are existing references from 13.7% to 38% (29, 30). This variability in protein content is attributed to genotype, environment, genotype × environment interaction, and the methods of determination. The heritability of protein content is moderate to high (31–33). However, the content and composition of pea protein are governed by complicated genetic mechanisms that involve multiple gene families (28).

The protein fraction in peas is primarily composed of globulins (legumin and vicilin) and albumins, which are storage proteins with relatively high nutritional value. Pea proteins are especially rich in the essential amino acids, lysine and arginine, although they are limited in sulfur-containing amino acids such as methionine and cysteine.

There are various references and reports on the relationship between pea protein content and seed characteristics, such as shape and color. According to Eliis et al. and Ren et al., (34, 35) wrinkled seeds have higher protein content and different protein composition compared to round seeds. However, other studies report that the protein content in peas was not affected by seed color and seed shape (30, 36).

Apart from protein content, protein composition is an essential factor for pea nutritive value. Pea protein excels compared to other plant proteins due to its high digestibility, comparatively lower occurrence of allergic reactions, and lack of adverse health issues. There are four main categories of pea protein: glutelin, prolamin, albumin, and globulin (37). Globulin makes up 55%−65% of the total protein content in peas, and is the energy storage protein (38), and is classified into 11S legumin and 7S vicilin (37). The legumin/vicilin ratio plays a significant role in the nutritional value and functionality of pea proteins (39). This ratio is about 2:1, with legumin composed of more sulfur-containing amino acids than vicilin per unit of protein (40). It has a positive correlation with total protein content, and even though it is mainly under genetic control, it is also affected by agronomic conditions (40). In this regard, Yang et al. (41) point out that breeding could alter this ratio.

Although the protein content of yellow peas tends to be lower (~23–25% dry matter) than that of soybeans and lupins, their protein quality and digestibility make them an attractive choice for dietary diversification.

According to Cervenski et al. (26), the average protein content in the tested material was 23.89%, and the total nitrogen content in the seeds ranged from 3.66 to 4.49 g per 100 g. In extruded gray pea products, the highest protein content was found in the sample without added grains or egg powder (26.9 ± 0.2 g 100 g^−1^), and the lowest (18.6 ± 0.5 g 100 g^−1^) was in the sample with the highest grain proportion (27).

Beyond their nutritional role, pea proteins exhibit functionalproperties, including emulsification, foaming, and gelling, makingthem highly suitable for food processing and formulation, particularly for developing meat analogs, dairy alternatives, andprotein supplements. Pea proteins are widely used in the food system, including for the encapsulation of bioactive compounds, the production of degradable films, and as an alternative to animal proteins. Some physicochemical characteristics of pea proteins closely resemble those of soybean proteins (5). The growing interest in pea proteins within the food industry reflects not only their nutritional value but also their allergen-free and sustainable nature compared to animal-derived or soy-based proteins. Importantly, pea protein is generally well-digested, making it more bioavailable and associated with fewer allergic reactions, enhancing its acceptance among diverse consumer groups.

#### 3.1.3 Lipids and other components

Although peas are not a primary dietary fat source, the quality of their lipid fraction complements their high fiber and protein content, further supporting their role as a low-fat, nutrient-dense, and heart-healthy food. Peas, like most temperate legumes, contain relatively low levels of total lipids, but contribute beneficial fatty acids and lipid-associated bioactives to the human diet (3). From a food science perspective, the low-fat content also contributes to extended shelf-life stability, since peas are less susceptible to lipid oxidation than oil-rich seeds and nuts.

On average, peas contain between 1.0 and 2.0 g of total fat per 100 g of dry seeds, with variation depending on cultivar, maturity, and agronomic conditions (42). While this represents a small fraction of their total dry matter, the qualitative composition of pea lipids is nutritionally significant. According to Cervenski et al. (26) fatty oil content in selected winter pea lines ranged from 1.48 to 1.89 g per 100 g. In the study by Strauta et al. (27), a greater fat content was found in the sample with onion flavor, up to 9.5 ± 0.5 g per 100 g, while the least was in the unseasoned sample, 0.6 ± 0.05 g per 100 g.

Despite the modest overall lipid concentration, the fatty acid profile of peas is nutritionally favorable, with unsaturated fatty acids predominating. The oil yield and fatty acid composition of Canadian legume varieties varied widely. The oil yield (% dry weight) ranged from 1.65 ± 0.10 for large green lentils to 8.39 ± 0.03 for Leader chickpeas. The samples were predominantly composed of unsaturated fatty acids, with polyunsaturated fatty acids (PUFAs) ranging from 46.81% to 66.88%, and monounsaturated fatty acids (MUFAs) ranging from 10.25% to 34.21%. The saturated fatty acid content ranged from 14.25% to 20.64%. Linoleic acid (18:2n−6) was the major fatty acid in the legumes, constituting 27.09% to 60.75% of the total lipids profile. It was followed by α-linolenic acid (18:3n−3), which ranged from 2.64% to 37.47%, oleic acid (18:1 cΔ9), ranging from 7.29% to 30.29%, palmitic acid (16:0), from 9.99% to 14.71%, stearic acid (18:0), from 1.25% to 3.40%, and myristic acid (14:0), from 0.30% to 3.46% (43).

These unsaturated fats are associated with cardioprotective effects, including the modulation of serum lipid profiles and the reduction of inflammation. Given the very low saturated fatty acid content, this further supports the health benefits of peas in populations with lipid sensitivity. The favorable ratio of unsaturated to saturated fats contributes to the overall cardiometabolic benefits of including peas in a balanced diet (1). In addition to fatty acids, peas contain lipid-soluble micronutrients such as small amounts of tocopherols (vitamin E) and phytosterols, which may contribute to antioxidant capacity and cholesterol-lowering effects (42).

While lipids are not the primary nutritional strength of peas, their high-quality fat profile complements the legume's overall health-promoting potential. It reinforces their role in plant-based, functional, and preventive nutrition strategies. Despite their relatively low lipid content, their unsaturated fatty acid profile and bioactive lipid compounds enhance their nutritional value and align with recommendations for diets aimed at reducing chronic disease risk.

### 3.2 Micronutrient content (vitamins, minerals)

In the context of modern nutrition, micronutrients, vitamins, and minerals required in small quantities play an essential role in maintaining optimal health, supporting growth, immunity, metabolism, and the prevention of chronic diseases (1, 4). While legumes are widely recognized for their macronutrient content, especially protein and fiber, their contribution as sources of bioavailable micronutrients is often underappreciated. Peas provide a range of essential micronutrients that complement their macronutrient profile and enhance their functional value in diverse diets. Peas contain a variety of water- and fat-soluble vitamins, along with key trace elements and macro-minerals, many of which contribute to physiological processes with public health relevance (3).

Among micronutrients, peas supply significant levels of folate (vitamin B9), vitamin K, vitamin C (in fresh forms), iron, magnesium, and phosphorus, all of which play essential roles in cellular metabolism, hematological function, and bone health. Peas are among the highest natural sources of folate, providing approximately 65–70 μg per 100 g of cooked peas (4, 44). Since folate is critical for DNA synthesis, red blood cell formation, and neural tube development, peas are a valuable food for pregnant women and in populations where neural tube defects remain a concern (4).

Additionally, yellow peas are rich in iron and lower in dietary fiber compared to other legumes, making them easier to digest and more suitable for a broader range of consumers. Jarecki and Migut, 2022 found that yellow peas contain adequate levels of essential micronutrients like phosphorus, potassium, and magnesium (45). Their iron content is particularly notable, enhancing their value in addressing iron deficiencies (45).

The tocopherol content obtained from lipophilic extracts varied among different legume varieties. For instance, in the yellow whole pea (Golden) variety, α-tocopherol was 2.40 ± 0.04 mg/g, γ-tocopherol was 54.17 ± 0.12 mg/g, δ-tocopherol was not detected (ND), and the total tocopherols were 57.00 ± 0.76 mg/g. In the yellow split pea variety, α-tocopherol was 2.30 ± 0.22 mg/g, γ-tocopherol was 46.14 ± 2.56 mg/g, δ-tocopherol was not detected (ND), and the total tocopherols were 48.44 ± 2.76 mg/g. In the green split pea variety, α-tocopherol was 2.13 ± 0.03 mg/g, γ-tocopherol was 50.39 ± 0.49 mg/g, δ-tocopherol was not detected (ND), and the total tocopherols were 52.52 ± 0.52 mg/g (43, 46).

The micronutrient composition of peas, especially when consumed as part of minimally processed or fresh, adds significant value to plant-based dietary patterns. In populations that reduce or avoid animal products, peas can help bridge common nutritional gaps, especially in iron, zinc, folate, and vitamin K.

Furthermore, their low cost, wide availability, and environmental sustainability make peas a strategic crop for combating micronutrient deficiencies globally, particularly among vulnerable or low-income populations.

### 3.3 Dietary fiber

Dietary fiber plays a central role in human nutrition, contributing to digestive health, regulating blood glucose levels, supporting metabolic regulation, and preventing chronic diseases such as cardiovascular disease and type 2 diabetes. Legumes are among the richest natural sources of dietary fiber, and peas in particular, offer a balanced profile of both soluble (pectin and oligosaccharides) and insoluble fiber (cellulose and hemi-cellulose), making them a valuable component of fiber-rich diets (47).

The total dietary fiber content in dried peas typically ranges from 14% to 26% of dry weight, while cooked peas provide approximately 4–7 g per 100 g, depending on the variety and preparation method. The intake of daily dietary fiber is about 25 g to 38 g per day in women and men (5).

In addition to traditional fiber classification, peas are rich in resistant starch, a form of carbohydrate that resists digestion in the small intestine and behaves similarly to dietary fiber in the colon. Resistant starch enhances glycemic control, promotes satiety, and supports the growth of beneficial gut microbiota, contributing to a healthy gut environment (48). The inclusion of pea-based ingredients, such as pea flour and protein isolates with retained fiber fractions, has been increasingly explored in functional food formulations aimed at improving fiber intake in modern diets (48).

### 3.4 Bioactive compounds (antioxidants, flavonoids, polyphenols)

Peas have emerged not only as an essential source of micro and macronutrients, such as protein, dietary fiber, and complex carbohydrates, but also as a reservoir of numerous phytochemicals with potential health-promoting properties (5, 46, 49). Among the most studied classes of phytochemicals in peas are phenolic compounds, flavonoids, saponins, and carotenoids, which exhibit antioxidant, anti-inflammatory, and anticarcinogenic effects (50, 51). These bioactive compounds, although not essential nutrients in the classical sense, can exert beneficial physiological effects that may contribute to the prevention and mitigation of various chronic diseases, including cardiovascular disorders, type 2 diabetes, certain types of cancer, and age-related degenerative conditions (4).

Peas are widely consumed worldwide in both fresh and dried forms, and they are frequently incorporated into dietary patterns associated with health benefits. Understanding the bioactive profile of peas becomes increasingly relevant not only from a nutritional standpoint but also for the development of nutraceuticals, functional ingredients, and health-promoting food products (52) (Table 4).

**Table 4:** Bioactive phytochemicals in peas and their bioactivities.

**Compound class**	**Primary components**	**Biological activities**
Phenolic compounds	Phenolic Acids: Gallic, ferulic, *p*-coumaric, syringic, caffeic, and chlorogenic acids.	Antioxidant, anti-inflammatory, antihypertensive.
Flavonoids	Flavonols: Quercetin, Kaempferol, Rutin. Flavones: Luteolin, Apigenin. Flavanols: Catechin, Epicatechin.	Strong antioxidant (chelating and radical scavenging), anti-inflammatory, anti-cancer (inhibition of cell proliferation).
Isoflavones	Genistein, Daidzein, Formononetin.	Estrogen-like activity, anti-cancer, cardioprotective.
Saponins	Triterpenoid glycosides (e.g., Soyasaponins).	Antioxidant, cholesterol-lowering (by complexing with bile acids), immunomodulatory, anti-cancer.
Carotenoids	beta-carotene, Lutein, Xanthophylls.	Antioxidant, provitamin A activity (beta-carotene), eye health (lutein).
Bioactive peptides	Small protein fragments (e.g., peptides rich in Glu, Asp, Gly, Pro, Leu).	Potent antioxidant, anti-hypertensive (ACE-inhibitory), anti-diabetic, anti-inflammatory.

**Figure 3:**
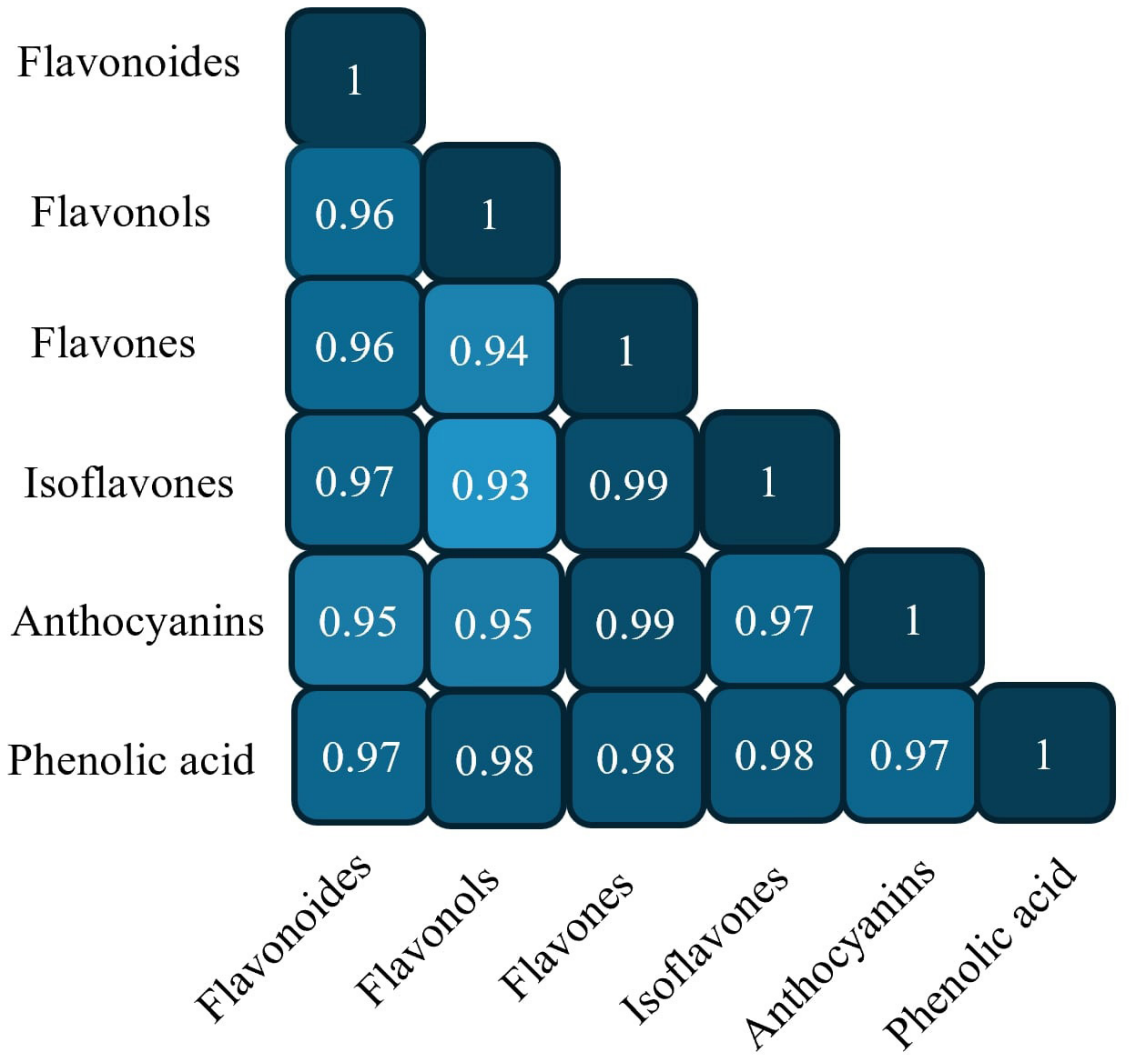
Correlation coefficients among phytochemical compounds in peas.

Phenolic compounds are among the most significant groups of bioactive constituents in peas, contributing to their antioxidant potential and nutritional value. Among these, total phenolic content (TPC) and total flavonoid content (TFC) are commonly used as indicators of antioxidant capacity (Figure 3). Numerous studies have demonstrated substantial variability in the phenolic profiles and antioxidant activities across different pea genotypes, influenced by seed characteristics such as color, shape, and seed coat composition (5, 46, 53, 54).

Peas with dark-colored seed coats have consistently been reported to contain significantly higher TPC than light-colored varieties. Similarly, round-seeded genotypes generally exhibit higher TPC compared to wrinkled seeds (5, 53, 55, 56). Phenolic acids represent the second most abundant class of polyphenols in peas, following flavonoids (57). Colored seed coats tend to accumulate higher levels of phenolic acids such as vanillic acid, gentisic acid, and protocatechuic acid. In contrast, white seed coats are more often associated with ferulic acid and p-coumaric acid. Additional phenolic acids identified in various pea tissues include vanillin acid, quinic acid, coumaroyl quinic acid, 5-feruloylquinic acid, 4-O-caffeoylquinic acid, trans-ferulic acid, trans-cinnamic acid, p-hydroxybenzoic acid, and 4-hydroxybenzoic acid, respectively (5).

Flavonoids identified in peas span several subgroups, including flavonols, flavones, isoflavones, flavanones, flavanols (flavan-3-ols), and anthocyanins. TFC has been shown to correlate with both seed coat color and morphology (5). These flavonoid compounds are known to exhibit potent antioxidant and anti-inflammatory properties and have been implicated in immune regulation (58, 59). Such bioactivities are primarily attributed to their radical scavenging capacity and general antioxidant potential (60).

An investigation of twelve pea accessions from southern Tunisia revealed substantial variability in protein, phenolics, and antioxidant capacity. Protein content ranged from 46.91 to 151.08 mg/g DW, while LC-ESI/MS analysis identified eight phenolic acids and nine flavonoids. Antioxidant activity, measured by DPPH and ABTS assays, was positively correlated with phenolic content but negatively with protein content. Genotypes with purple flowers, brown seed coats, and wrinkled seeds exhibited the highest TPC and antioxidant values (61).

Chen et al. (50) analyzed ten pea varieties and reported TPC ranging from 0.66 to 2.66 mg/g, and TFC from 0.74 to 1.88 mg/g, with the highest phenolic content observed in variety ZW.8. The TFC values were notably higher than those reported for soybeans (50, 62). Similarly, peas grown in Nepal showed TPC of 33.14 ± 0.50 mg GAE/g and TFC of 137.12 ± 4.3 mg QE/g (57). Zhao et al. (63) assessed 75 pea varieties, revealing TPC values ranging from 0.27 to 1.95 mg GAE/g dry weight. Twenty-two varieties had above-average phenolic levels, and TFC varied from 0.53 to 5.08 mg rutin equivalents/g dried weight. Yellow peas exhibited TPC values of 0.85–1.14 mg/g and TFC of 0.09–0.17 mg/g, while green peas showed slightly lower phenolic and flavonoid content.

Qualitative and quantitative analysis of acetone extracts from pea seeds led to the identification of 25 phenolic compounds, including five not previously reported in peas (rosmarinic acid, morin, naringin, naringenin, chrysin). Dueñas et al. (64) found high levels of hydroxybenzoic acids in dark-colored seeds, while Troszynska and Ciska (56) identified vanillic, syringic, and o-coumaric acids. Stanisavljević et al. (65), on the other hand, reported exceptionally high epigallocatechin content in specific dark-colored cultivars such as Aslaug, Assas, Dora, Poneka, MBK 168, and MBK 173 (65).

Advanced metabolite profiling using UHPLC-LTQ-Orbitrap MS identified 41 phenolic compounds, including rutin, galangin, morin, naringin, hesperetin, and pinocembrin (65). Notably, 10 flavonol glycosides previously unreported in European cultivars were detected. Varieties MBK 168 and MBK 173, characterized by dark seed coats, showed high TPC and antioxidant activity, while light-colored varieties MBK 88 and MBK 90 exhibited superior metal-chelating activity. The developmental stage also significantly impacts phenolic composition and antioxidant capacity. A study on six garden pea varieties across ripening stages found that the highest antioxidant activity was recorded in the array ‘Jumbo' (1,179.99 ± 28.08 mg/kg). At the same time, ‘Premium' showed the lowest (674.51 ± 26.54 mg/kg). The variety ‘Flavora' was identified as optimal for human nutrition, with significant differences observed among different maturity groups. In a comparative study of 14 Canadian legume varieties, green peas exhibited the lowest TPC (1.16 ± 0.08 mg GAE/g DW), while large green lentils showed the highest (7.45 ± 0.69 mg GAE/g DW). Dehulling markedly reduced phenolic content, indicating that the seed coat plays a central role in phenolic accumulation. Among peas, yellow whole, yellow split, and green split varieties showed TPC values of 1.38 ± 0.06, 1.21 ± 0.06, and 1.16 ± 0.07 mg GAE/g DW, respectively (43).

Although not a phenolic component, carotenoid content is another group of bioactives of interest in peas. In Canadian yellow and green pea varieties, total carotenoid content ranged from 21.17 ± 1.53 mg/g to 29.61 ± 4.46 mg/g, with lutein being the dominant carotenoid (43, 66). These findings further support the classification of peas as a functional food, given their contribution to antioxidant defense and their potential health benefits.

## 4 Health benefits of peas

Epidemiological studies have consistently shown that higher consumption of legumes, including peas, is associated with a reduced risk of non-communicable diseases such as cardiovascular disease, metabolic syndrome, type 2 diabetes, and certain types of cancer (4, 67). As a rich source of plant-based protein, dietary fiber, and vitamins, peas are a functional food with the potential to support disease prevention and health maintenance. As previously mentioned, the protective effects are attributed not only to their favorable nutrient profile but also to the presence of a diverse array of bioactive compounds, including polyphenols, saponins, carotenoids, and other bioactive peptides. These compounds could exert a range of physiological effects, including antioxidant, anti-inflammatory, antihypertensive, hypoglycaemic, and lipid-lowering actions (68–70). The significance of phenolics is better understood due to their extensive range of beneficial properties (69–72). The soluble and insoluble fibers present in peas play a critical role in improving glycemic control, enhancing satiety, and supporting gut microbiota diversity, thereby contributing to metabolic health. In addition, pea protein has demonstrated beneficial effects on muscle maintenance, blood pressure regulation, and postprandial glucose response, making it a valuable alternative in plant-based and clinical nutrition (1, 54).

In summary, peas possess multiple biological properties, including regulation of conditions related to metabolic syndrome, modulation of gut microbiota, antioxidant effects, anti-inflammatory properties, antihypertensive effects, obesity management, anticancer properties, anti-fatigue effects, antidiabetic properties, antimicrobial effects, anti-renal fibrosis effects, immunomodulatory activities, and prevention of osteoporosis (49). Furthermore, recent preclinical and clinical studies have highlighted the role of peas in modulating key biomarkers of inflammation and oxidative stress, improving endothelial function, and supporting immune resilience (59, 73). These findings underscore the potential of peas not only as a component of a balanced diet but also as a functional food with applications in the prevention and adjunctive management of chronic diseases. The following sections will provide a detailed examination of some of the mentioned effects of pea consumption, as well as some pea by-products on various aspects of human health.

### 4.1 Antioxidant activities

Numerous studies have demonstrated that peas and their by-products exhibit significant antioxidant activity, primarily due to their diverse bioactive components, including polyphenols, phenolic acids, polysaccharides, and peptides. A higher polyphenol content is generally associated with a greater antioxidant capacity, as previously reported for various pea cultivars (49). Dark-colored pea seeds (e.g., brown and dark purple) exhibit higher concentrations of polyphenolic and flavonoid compounds than lighter-colored seeds, resulting in enhanced antioxidant potential. This activity is mainly linked to the presence of compounds such as gallic acid, epigallocatechin, naringenin, and apigenin.

Comparative analyses performed by Koley et al. (60), on underutilized and commonly cultivated leguminous vegetables, including garden pea and edible-podded pea (*Pisum sativum* var. macrocarpon), confirmed notable antioxidant capacity in bivariate when evaluated using standard *in vitro* assays (FRAP, CUPRAC, DPPH, and TEAC). Garden peas were particularly recognized for their tender seeds, while edible-podded peas demonstrated relevance for whole pod consumption (1, 55). Despite some variability in results due to methodological differences across studies, the overall evidence consistently supports peas as an essential dietary source of antioxidants. This conclusion is further reinforced by the findings of Kabir et al. (74), who reported that peas exhibited the highest flavonoid content (17.64 mg catechin equivalent/g dry extract) and the best reducing power (OD = 1.11) among 12 commonly consumed leafy vegetables in Bangladesh. The strong correlation (*r*^2^ = 0.87) between flavonoid content and reducing power indicates that their flavonoid composition primarily drives the antioxidant activity potential of peas (74).

#### 4.1.1 Immune system support

Oxidative damage has been proven to cause many diseases, such as type 2 diabetes, inflammation, and certain cancers, and is driven by an excess of reactive oxygen and nitrogen species (75–77). Antioxidants, found in foods, can not only inhibit enzymes associated with ROS (reactive oxygen species) but also protect cells from oxidative damage and boost the immune system (51, 55). The main feature of phenolic food compounds is antioxidant activity, which is considerable for health preservation (75–77). When foods that strengthen the immune system are incorporated into the daily diet, the body receives the necessary nutrients. In this way, nutrition increases biological defense mechanisms, slows down the aging process, controls physical and mental disorders, and prevents and cures certain diseases (59, 73). According to growing scientific studies of naturally occurring vegetables and fruits, green peas are among the products with the highest antioxidant potential (62). The phenolic compounds found in peas are considerable active ingredients that are thought to have many health benefits beyond the ones listed above (50).

### 4.2 Potential in managing metabolic syndrome-blood sugar control, diabetes management, and weight management

The numerous biological activities and health-promoting effects of peas are closely associated with their nutritional and bioactive composition. Rich in dietary fibers, plant-based proteins, resistant starches, polyphenols, and micronutrients, peas are considered a promising dietary component for mitigating metabolic dysfunctions related to metabolic syndrome diseases (49, 78). With a typically low-to-medium glycemic index (below 60), peas can help improve glycemic control and partially replace high-GI foods in the diet. Being gluten-free, they are also suitable for individuals with celiac disease (49).

Metabolic syndrome is characterized by a combination of metabolic disorders, including obesity, insulin resistance, dyslipidemia, hypertension, and central obesity, all of which significantly elevate the risk of developing cardiovascular diseases (79). A growing body of evidence supports the ability of peas and their components to modulate these risk factors. Several *in vitro* and *in vivo* studies have demonstrated reductions in waist circumference, fasting glucose, and blood pressure following regular pea consumption, highlighting their potential in improving overall metabolic health (1, 39, 80). The underlying mechanisms include enhanced insulin sensitivity, regulation of lipid metabolism, modulation of adipokine secretion, and improved appetite control through ghrelin and peptide signaling (4, 81, 82). Resistant starch in peas further slows carbohydrate digestion, promoting glycemic stability and reducing insulin resistance (83, 84).

Beyond glycemic regulation, peas exert beneficial effects on cardiovascular health by improving lipid profiles, lowering total cholesterol, LDL, and triglycerides, while elevating HDL levels (8, 85). Their fiber and prebiotic components also contribute to gut microbiota diversity, reducing systemic inflammation and supporting metabolic balance (86). Peptides derived from pea protein hydrolysates exhibit notable antihypertensive activity through ACE and renin inhibition, while hypolipidemic and antioxidant effects have been confirmed both *in vitro* and *in vivo* (8).

Recent studies also emphasize that pea by-products, such as pods, display bioactive potential and should not be overlooked. Inagaki et al. (87) reported that autoclaved pea pod extract significantly reduced serum triglyceride and cholesterol levels in rats fed a high-sucrose diet, linked to enhanced lipid excretion, pancreatic lipase inhibition, and prebiotic activity via bifidobacteria proliferation. Similarly, methanolic pea pod extract demonstrated antidiabetic and antihyperlipidemic effects in alloxan-induced diabetic mice, improving glucose metabolism, liver and kidney function, and antioxidant enzyme activity (88).

At the molecular level, specific pea-derived proteins and glycoproteins have shown promise as natural therapeutics. Pea albumin supplementation in high-fat diet-fed mice improved glucose tolerance, insulin sensitivity, and hepatic lipid metabolism, while suppressing inflammation and adipogenesis (89). Pea glycoprotein PGP2 reduced fasting glucose and improved insulin secretion in diabetic mice through activation of the IRS–PI3K–Akt–GLUT1 pathway, demonstrating strong antidiabetic potential (89). Qin et al. (78) further identified three glycoprotein fractions (PGP1–PGP3) with inhibitory effects on α-glucosidase and α-amylase, with PGP2 exhibiting the most significant activity due to its low molecular mass and high arabinose and glucose content.

Collectively, these findings confirm that peas and their by-products can modulate key metabolic pathways, including glucose and lipid metabolism, oxidative stress, and inflammation, thereby reducing risk factors associated with metabolic syndrome. However, most available data derive from *in vitro* and animal studies, and further clinical research is essential to validate these effects in humans and to optimize the utilization of pea-derived bioactives in functional foods and nutraceuticals.

### 4.3 Digestive health

Peas are an excellent source of both soluble and insoluble dietary fiber, contributing significantly to digestive health, promoting regular bowel movements, and supporting overall gut function (90). Soluble fiber ferments in the colon, producing short-chain fatty acids (SCFAs) such as butyrate, which nourish colonocytes, maintain mucosal integrity, and form a gel-like substance in the gut that can aid in slowing digestion and improving nutrient absorption. This can also help manage blood sugar levels and reduce the risk of digestive disorders, such as constipation or irritable bowel syndrome (IBS). Insoluble fiber adds bulk and accelerates transit time, effectively reducing constipation. Additionally, the fermentation of pea fiber supports a beneficial gut microbiota composition, which could be linked to reduced inflammation and improved immune function, promoting a healthy microbiome (86). The administration of pea albumin in the study of Liu et al. (89) has been demonstrated to restore the composition of the gut microbiota to a state more closely resembling that observed under normal fat diet conditions. This process has been shown to selectively promote the growth of beneficial gut bacteria, such as *Akkermansia* and *Parabacteroides*. The study's findings demonstrated that pea albumin negatively modulated lipid accumulation by suppressing adipogenesis and promoting fatty acid oxidation. This finding further substantiates its role in the management of obesity. The study highlights the potential of pea albumin as a functional ingredient for preventing obesity and improving metabolic health through multiple mechanisms, including modulation of lipid metabolism and gut microbiota composition. The study of Guo et al. (91) explores the therapeutic potential of pea-based products, green pea hull (GPH) extracts, in managing ulcerative colitis (UC), with a particular focus on their antioxidant and gut-modulating properties. Using a DSS-induced mouse model, the researchers demonstrated that GPH extracts could alleviate colitis symptoms by enhancing colonic function and reducing inflammation. These effects are primarily mediated through the activation of the Keap1-Nrf2-ARE signaling pathway, which plays a key role in regulating oxidative stress and inflammatory responses. GPH ex-tracts are rich in phenolic compounds, especially quercetin, kaempferol, and catechin, which contribute to their bioactivity. Additionally, the extracts favorably modulated the gut microbiota, promoting beneficial bacteria such as *Lactobacillaceae* and *Lachnospiraceae* and increasing levels of short-chain fatty acids (SCFAs), which are essential for intestinal health. Collectively, these findings highlight GPH extracts as a promising functional food ingredient and sustainable dietary strategy for UC management, derived from an underutilized pea by-product.

### 4.4 Cardiovascular health

As was highlighted in the previous sections, peas play a crucial role in addressing key risk factors for heart disease, including cholesterol levels, blood pressure, weight management, and inflammation, acknowledged by their rich nutrient profile, which includes fiber, protein, antioxidants, and essential vitamins (49). Regular consumption of peas as part of a balanced diet can help improve blood pressure and reduce cholesterol levels. The high fiber content in peas can help lower cholesterol levels by binding to bile acids and preventing their reabsorption, which in turn may reduce the risk of heart disease. Soluble fiber binds bile acids, promoting their excretion and forcing the liver to use more cholesterol to synthesize new bile acids (92). The antioxidants in peas, such as flavonoids and vitamins, help reduce inflammation and oxidative stress, both of which are associated with cardiovascular problems (68). Peas contain phytosterols, flavonoids (e.g., kaempferol), and dietary fibers, which together could contribute to improved lipid profiles and reduced LDL cholesterol. Furthermore, the antioxidant properties of polyphenols present in peas help reduce oxidative stress and endothelial dysfunction, which are key contributors to atherosclerosis. Additionally, peas are a good source of plant-based protein, which can be a heart-healthy alternative to animal-based proteins that are often higher in saturated fats. The afore-mentioned and numerous additional mechanisms, attributable to the compositional profile of peas, act synergistically to promote cardiovascular health when peas or pea-based products are incorporated into the diet.

### 4.5 Bone health

Peas are rich sources of vitamins and minerals, including vitamin K, magnesium, and calcium, which are vital for bone mineralization, remodeling, and maintaining bone density (38). A 100 g serving of peas provides a nutritionally significant profile critical for maintaining bone density: 8% of daily calcium, 39% of magnesium, 45% of manganese, and 43% of phosphorus relative to recommended daily values (3). The mineral composition of peas demonstrates a pivotal role in bone metabolism. Field peas contain essential minerals critical for bone health, including potassium (97–99 mg/100 g), calcium (9–11 mg/100 g), magnesium (5–7 mg/100 g), and trace minerals such as copper, selenium, and boron (1, 4, 88, 93). These minerals and vitamins exhibit synergistic interactions that modulate bone density and structural integrity and support the physiological mechanisms of bone health. Notably, mineral concentrations can vary significantly across cultivars, with calcium reaching up to 96. 25 μg/g and magnesium hitting 94.59 μg/g, depending on cultivation regions (7). The nutritional attributes of peas extend beyond their fundamental mineral content. Vitamin K, a critical micronutrient for bone metabolism, facilitates biochemical interactions with proteins such as osteocalcin and matrix Gla-protein (MGP), which are fundamental to bone mineralization and calcium sequestration (80, 81). Although peas are not considered a predominant source of vitamin K, their comprehensive mineral profile significantly contributes to the overall framework of bone health. Vitamin K (especially K1 found in green vegetables like peas) is essential for the carboxylation of osteocalcin, a protein necessary for binding calcium in the bone matrix (94, 95).

Since peas represent a compelling nutritional source with significant potential to support bone health through their intricate mineral and vitamin composition, processing methodologies can potentially enhance their nutritional bioavailability. Research published by Alonso et al. (44) demonstrated that thermal extrusion processing at 150 °C minimally alters the elemental composition of calcium, magnesium, and phosphorus while substantially enhancing mineral absorption in preclinical animal models. This improvement is hypothetically attributed to the degradation of antinutrient compounds such as phytates.

Epidemiological investigations underscore the potential of peas for bone health, with emerging evidence linking increased calcium and vitamin K intake to reduced fracture risk, particularly among older populations (96, 97). Supplementation studies exploring vitamins K, D, and calcium have produced inconsistent results regarding overall bone density, necessitating a rigorous and systematic investigation (95–102). As peas stand out as a natural, nutrient-dense option for supporting bone density due to their diverse mineral composition, despite their promising nutritional potential, existing research highlights the need for more comprehensive, longitudinal clinical trials.

### 4.6 Anti-inflammatory effects vs. role in reducing inflammation

Chronic diseases are often driven by inflammation, and an intriguing area of research has concentrated on the potential anti-inflammatory properties of peas. Chronic inflammation contributes to various health issues, including diabetes, cardiovascular disease, and arthritis (79). However, it can be mitigated by consuming bioactive compounds sourced from peas (72). Polyphenols found in peas inhibit the NF-κB signaling pathway, which reduces the production of pro-inflammatory cytokines such as TNF-α and IL-6 while boosting levels of IL-10, thus promoting immune regulation and decreasing the inflammatory process (67, 103, 104). In a study by Schoeny et al. (105), researchers explored the connection between the availability of primary inoculum and the severity of Ascochyta blight in pea plants. Their findings uncovered a significant relationship between immune modulation and reduced systemic inflammation, suggesting that understanding these dynamics could improve disease management strategies. In a related line of research, Fristensky et al. (106) investigated the complex changes in gene expression that occur in peas during interactions with the pathogenic fungus *Fusarium solani*. This study revealed critical molecular responses that correlated with reduced levels of inflammatory markers, especially in contexts where the pea varieties displayed disease resistance. Together, these studies highlight the intricate relationship between plant immunity and inflammation, demonstrating the potential of peas to alleviate chronic inflammatory conditions in humans. Lignans in peas may help suppress the action of inflammatory enzymes, particularly COX and LOX, which are involved in the synthesis of pro-inflammatory mediators (52). Additionally, the dietary fiber in peas promotes gut health by encouraging the growth of anti-inflammatory bacteria, which helps reduce systemic inflammation (86, 107). Clinical studies have shown that consuming peas can enhance outcomes in inflammatory conditions like inflammatory bowel disease (IBD) and rheumatoid arthritis, further supporting their anti-inflammatory benefits (1, 39, 108). Peas are acknowledged as a source of anti-inflammatory phytonutrients, including coumarin and saponins, which modulate inflammatory cytokines such as IL-6 and TNF-α. Regular consumption may help reduce chronic low-grade inflammation associated with cardiovascular and metabolic diseases (5).

### 4.7 Peas in disease prevention (antioxidant properties and cancer prevention)

Peas are widely appreciated not only for their nutritional value but also as significant sources of antioxidants (109). s. These compounds play a crucial role in protecting cellular integrity by neutralizing free radicals and mitigating oxidative stress, unstable molecules that can cause oxidative damage to cells and tissues (60, 62). Chronic oxidative stress is implicated in the development of various diseases, including cancer (49, 76, 80). Highlighting the importance of these protective mechanisms. In plants, such as peas, oxidative stress triggers complex defense responses, including the induction of specific mRNAs and enzymatic activities, such as malate dehydrogenase, that regulate metabolic pathways to maintain cellular homeostasis (110, 111). These processes reflect conserved biochemical strategies shared with human cells, suggesting that the antioxidant and stress-mitigating properties of peas may not only support plant health but also have implications for human disease prevention, including cancer. Consequently, the health benefits of peas extend beyond classic nutrition, encompassing molecular mechanisms that enhance resilience against oxidative damage.

Given their diverse composition of bioactive compounds, peas exhibit remarkable antioxidant and anticancer potential. Phenolic acids such as ferulic and caffeic acid efficiently neutralize reactive oxygen species (ROS), protecting cellular macromolecules from oxidative damage (53, 112). Numerous studies have demonstrated the significant relationship between diet and cancer prevention, highlighting the protective effects of legumes like peas (113). The remarkable antioxidant capacity of peas contributes to the neutralization of free radicals and the attenuation of inflammation, two central mechanisms in cancer development and progression (114, 115).

Flavonoids can significantly inhibit lipid peroxidation, reduce ROS-induced cellular damage, and prevent genetic mutations that can lead to tumorigenesis (46, 116). Furthermore, galactolipids found in peas suppress angiogenesis, a crucial process in tumor growth, particularly in hormone-sensitive cancers such as breast cancer (80). Green peas contain pigments such as carotenoids (like lutein and zeaxanthin), which exhibit antioxidant and antimutagenic activity by neutralizing free radicals, reducing DNA damage, and modulating detoxification enzymes (43). Additionally, saponins present in peas can induce apoptosis in cancer cells by disrupting signaling pathways and limiting metastatic potential. Overall, regular consumption of peas is beneficial due to their combined role in reducing oxidative stress and lowering cancer risks (1, 4, 53, 117).

## 5 Toxins and antinutrients in peas

**Figure 4:**
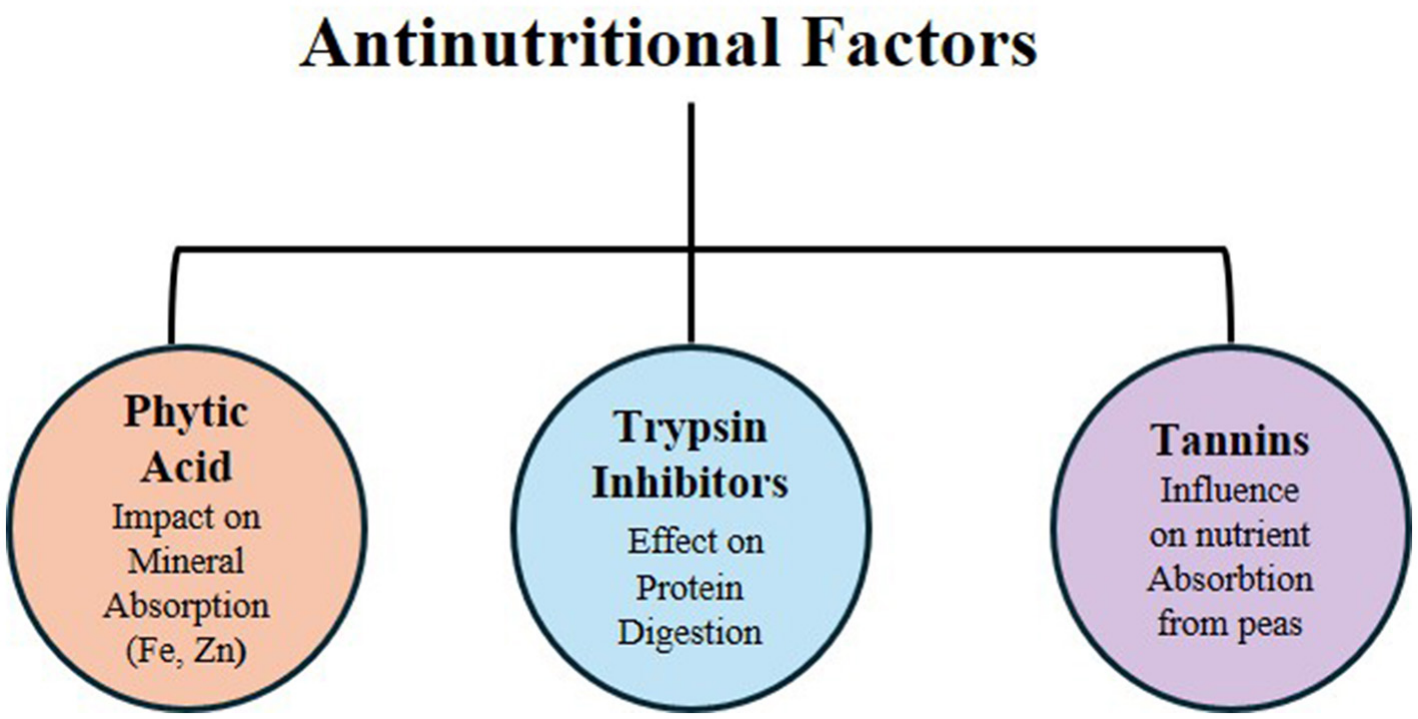
Antinutritional factors of peas.

The use of grain legumes as food sources is restricted by antinutritional factors, which could have harmful effects on human health (118). These factors are naturally produced as secondary metabolites that protect the plant against biological stresses, such as pest attacks. The main antinutrients are tannins, phytic acid, cyanogenic glycosides, saponins, oxalates, biogenic amines, lectins, protease inhibitors, and amylase inhibitors. The most concerning metabolites in peas are phytic acid, tannins, and trypsin inhibitors (119). These components interfere with the nutritional value of foods by reducing mineral absorption and protein digestibility, and by causing toxicity and health disorders when present at high concentrations. In previous sections, it was established that antinutrients have a strong negative relationship with micronutrient bioavailability, as higher levels of antinutrients reduce the availability or absorption of minerals and can lead to nutrient deficiency or malnutrition (Figure 4).

### 5.1 Antinutritional factors

#### 5.1.1 Phytic acid and its impact on mineral absorption (iron, zinc)

Phytates, which serve as reservoirs for phosphate and minerals in seeds, may also affect the absorption of Zn and Fe from crops (120, 121), potentially limiting their nutritional value. Phytic acid forms insoluble complexes with minerals (copper, iron, and zinc), resulting in a reduction in their absorption in the human gastrointestinal tract (85). Iron deficiency is a primary global health concern, affecting infants, children, and women, leading to child and maternal mortality, decreased mental development, and increased susceptibility to infectious diseases. The leading cause of Fe deficiency is prolonged consumption of a non-diverse plant-based diet low in bioavailable Fe (66). To improve Fe and Zn bioavailability, several strategies have been used to reduce phytate content in foods. Additionally, low phytic acid crops have been produced through plant breeding, further enhancing the effectiveness of these strategies (122, 123). Liu et al. (124) showed that pea varieties with non-pigmented seed coats (i.e., low in polyphenols) had seven times greater Fe bioavailability than those with pigmented seed coats. High concentrations of polyphenolic compounds in the seed coats of Fe-biofortified common bean and black bean limited iron bioavailability (120, 125, 126). These results suggest that higher Fe concentration could be associated with higher Fe bioavailability in pea varieties with non-pigmented seed coats. Low-phytate pea lines have higher iron bioavailability than standard varieties (127). Previous studies suggest that biofortified crops with high iron content can improve iron status in iron-deficient individuals (121, 125, 128). However, recent research indicates that the phytic acid/Fe molar ratio is crucial for predicting iron absorption from digested food, with a 10:1 ratio resulting in the most significant inhibition of iron bioavailability. A recent study found that despite comparable iron levels among all pea varieties, the intake of lower-phytate peas moderately enhanced iron status and storage, as evidenced by higher hepatic ferritin levels in lower-phytate groups compared to high-phytate and no-pea diets (129). Lower-phytate pea-based diets also showed elevated expression of ferroprotein, an iron exporter that facilitates iron transfer across enterocytes (130). Although hemoglobin levels were not significantly increased in lower phytate pea groups, notable changes were found in whole body hemoglobin iron, indicating an improvement in iron status (128, 131–133).

#### 5.1.2 Trypsin inhibitors and their effect on protein digestion

Trypsin inhibitors significantly inhibit the activity of the pancreatic enzymes trypsin and chymotrypsin, which are among the most crucial serine proteases. This reduces the digestion and absorption of dietary proteins, even in the presence of high levels of digestive enzymes (134). Inhibition of these enzymes is crucial, as excess protein intake can lead to weight gain (135), because excess amino acids are used to synthesize acetyl-CoA, a precursor of triglycerides stored as body fat. In addition, excess amino acids can be used as substrates for glucose production during gluconeogenesis (136). The trypsin inhibitors of legumes are divided into 2 families according to their molecular size: Kunitz (KTIs) (~20 kDa), which are predominantly active against trypsin, and Bowman-Birk (BBTIs) (~20 kDa), which are active against trypsin and chymotrypsin (137). Thermal treatments are widely accepted as the most effective method for improving the nutritional value of legume seeds by inactivating thermostable antinutritional factors, particularly trypsin inhibitors. These treatments promote the breakage of intermolecular bonds, thereby altering the active site conformation. Some methods used in thermal treatments include cooking, boiling, autoclaving, microwaving, and roasting. For pea seeds, microwaving (2,450 MHz for 4 min), boiling (100 °C for 20 min), and pressure cooking (120 °C for 10 min) resulted in total inactivation of trypsin inhibitors, improving *in vitro* protein digestibility by 6.53%, 6.28%, and 2.72%, respectively (137). Nonetheless, despite the efficacy and rapidity of these processes, it has been noted that temperatures exceeding 80 °C may compromise specific essential nutrients, including lysine, sulfur amino acids, and heat-sensitive vitamins (138–141). As a preventive agent, protease inhibitors have been shown to inhibit the proliferation of preadipocytes, suggesting a potential to combat obesity (142). Serine protease inhibitors have also been associated with boosting the immune system and blood development (143–145). Synthetic inhibitors of digestive enzymes have been developed for various purposes; however, they often have adverse effects, ranging from diarrhea to hepatotoxicity (146). Given the global prevalence of obesity and type 2 diabetes mellitus (T2DM), the identification of alternative enzyme inhibitors is essential. Compounds from natural sources (e.g., dietary components) are more desirable because they are reported to have a lower risk of adverse side effects than synthetic inhibitors (8).

#### 5.1.3 Tannins and their influence on nutrient absorption from peas

Legumes are a rich source of essential nutrients, such as minerals and dietary proteins. They are a healthier alternative to animal proteins, as they are not associated with cardiovascular disease, but they also contain significant amounts of tannins. Tannins can interfere with the protein digestive process and hinder the efficient utilization of nutrients (147–150). The effects of bean tannins on protein digestibility may be related to their ability to form stable and insoluble complexes with dietary proteins (79, 151–153). The tannin content of certain beans can be reduced by soaking in various solutions (154), possibly due to the leaching of polyphenols into the soaking water (150, 155). Therefore, these polyphenolic compounds, which are mostly water-soluble and found in the seed coat, can be reduced by pre-processing methods like soaking, which can help reduce the level of water-soluble/leachable antinutritional factors, such as tannins (156).

Lectins are metabolites that can be found in cereals, legumes, and nuts, which can attach to red blood cells, bind to carbohydrates, and attach to the lining of the gut/small intestine (157, 158). Lectins can agglutinate red blood cells, interfere with carbohydrate digestion, and cause mineral and nutrient deficiency (158).

Tannins, saponins, and lectins can also interfere with nutrient absorption, but through a different mechanism. Instead of forming a complex system with micronutrients, they bind to the lining of the human gut and promote inflammation, thereby preventing the absorption of nutrients and minerals (158–160). Therefore, these antinutrients should be reduced (if not completely removed) as they can cause numerous and severe health problems associated with deficiencies of minerals and nutrients (160, 161). Although lectins are resistant to enzymatic digestion in the gastrointestinal tract, they can be removed from foods by various processes. For example, soaking, autoclaving, and boiling cause irreversible denaturation of lectins. Boiling legumes for 1 hour at 95 °C reduced hemagglutinating activity by 93.77%−99.81% (162). Microwave ovens, on the other hand, are not an effective method for lectin deactivation. Though microwaving destroyed hemagglutinins in most legume seeds, it did not significantly affect lectins in common beans (162). Additionally, fermentation over 72 h has been demonstrated to destroy almost all lectins in lentils (*Lens culinaris*) (162).

Saponin can be found in legumes, tea leaves, and Allium species, seeds that inhibit the activities of digestive enzymes, disrupt the membrane cholesterol of erythrocytes (160, 163, 164), and decrease nutrient absorption, indigestion disorders, and hemolysis (160, 165).

### 5.2 Methods to reduce antinutrients and toxins

Due to the presence of various anti-nutritional factors in legumes such as peas, their nutritional quality and beneficial effects may be limited. Different processing techniques have been applied to peas, improving their functional properties and expanding their applications in the food industry. Processing methods, such as cooking, soaking, roasting, boiling, pressure cooking, milling, drying, and sprouting, are effective in decreasing antinutritional factors.

Extrusion of different blends of rice, carob (*Ceratonia siliqua*) fruit, and green pea significantly reduced the myo-inositol hexakisphosphate (phytic acid) or IP6 (5.7%−30.9%) contents, while the lectin contents were reduced by 50%−100%, and the protease inhibitors were eliminated (166, 167).

Cooking also affected the contents of bioactive compounds in peas, such as reducing total polyphenols by 48%−70%, reducing total saponins by 14%−30%, and reducing total oligosaccharides by 20% (168). On the other hand, cooking can improve the nutritional quality by inactivating or reducing the level of anti-nutritional factors (169), could increase free phenolic acids in peas and reduce bound phenolic acids; the carotenoid content in peas increased significantly after cooking treatment.

Various previous studies have confirmed that fermentation is one of the best methods for reducing anti-nutritional factors in foods compared to other methods. However, germination followed by fermentation also showed promising results for reducing the level of antinutrients in foods. Microbial fermentation activates many endogenous enzymes, like phytase, which generally degrade phytate in the food. Therefore, the quality of food crops like cereals and grains can be improved by subjecting them to various processing methods, especially germination and fermentation (158).

Soaking is a necessary step that precedes other food processing treatments, such as cooking, microbial fermentation, and germination. Soaking does not significantly alter the chemical composition of pea flours but does affect their physicochemical properties and antinutritional factors. Soaking could disrupt the protein and fibrous matrix around starch granules in yellow peas, leading to greater swelling during gelatinization and thereby increasing the gelatinization viscosity of yellow pea flours. Soaking could increase the protein solubility of yellow pea flours to some extent, possibly due to proteolytic enzyme breakdown of proteins. Soaking could also significantly reduce the levels of lectins and oxalates in peas, but did not affect phytic acid levels. Soaking the seeds of soya bean, green pea, chickpea, lentil, broad bean, and common bean in distilled water significantly decreased the contents of lectins and total and soluble oxalates (169). Compared to soaking, the cooking process was more efficient in reducing the levels of lectins, oxalates, IP6, saponins, and tannins in legume seeds. Milling is a process that utilizes mechanical force to break down particles into smaller pieces or fine particles, affecting *in vitro* starch and protein digestion properties (169).

However, the selection of processing methods should consider the balance between nutrient retention and the reduction of antinutritional compounds. Excessive heat or prolonged processing can lead to the loss of essential amino acids, vitamins, and phenolic compounds. In contrast, milder treatments, such as fermentation and germination, effectively reduce phytates, tannins, and trypsin inhibitors while improving protein digestibility and mineral bioavailability. From a technological perspective, optimizing these processes is essential to developing pea-based food products with improved nutritional quality and functional properties suitable for industrial applications.

## 6 Comparison of peas with other common legumes and unique properties of peas; pea-based products, and the application of peas; environmental benefits; market perspectives; current gaps in research and future directions

**Figure 5:**
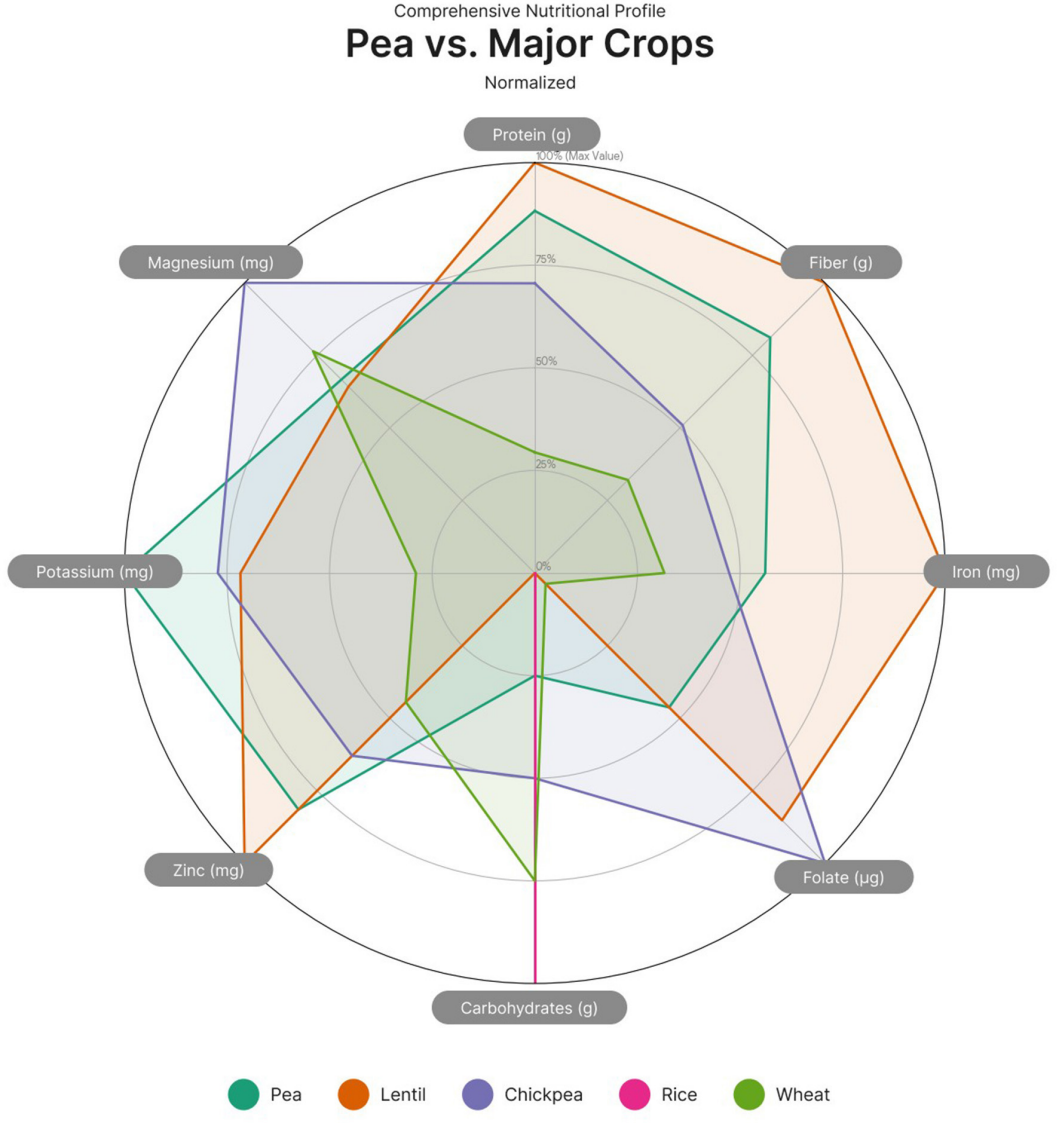
Comparison of peas' nutritional components with major crops.

**Figure 6:**
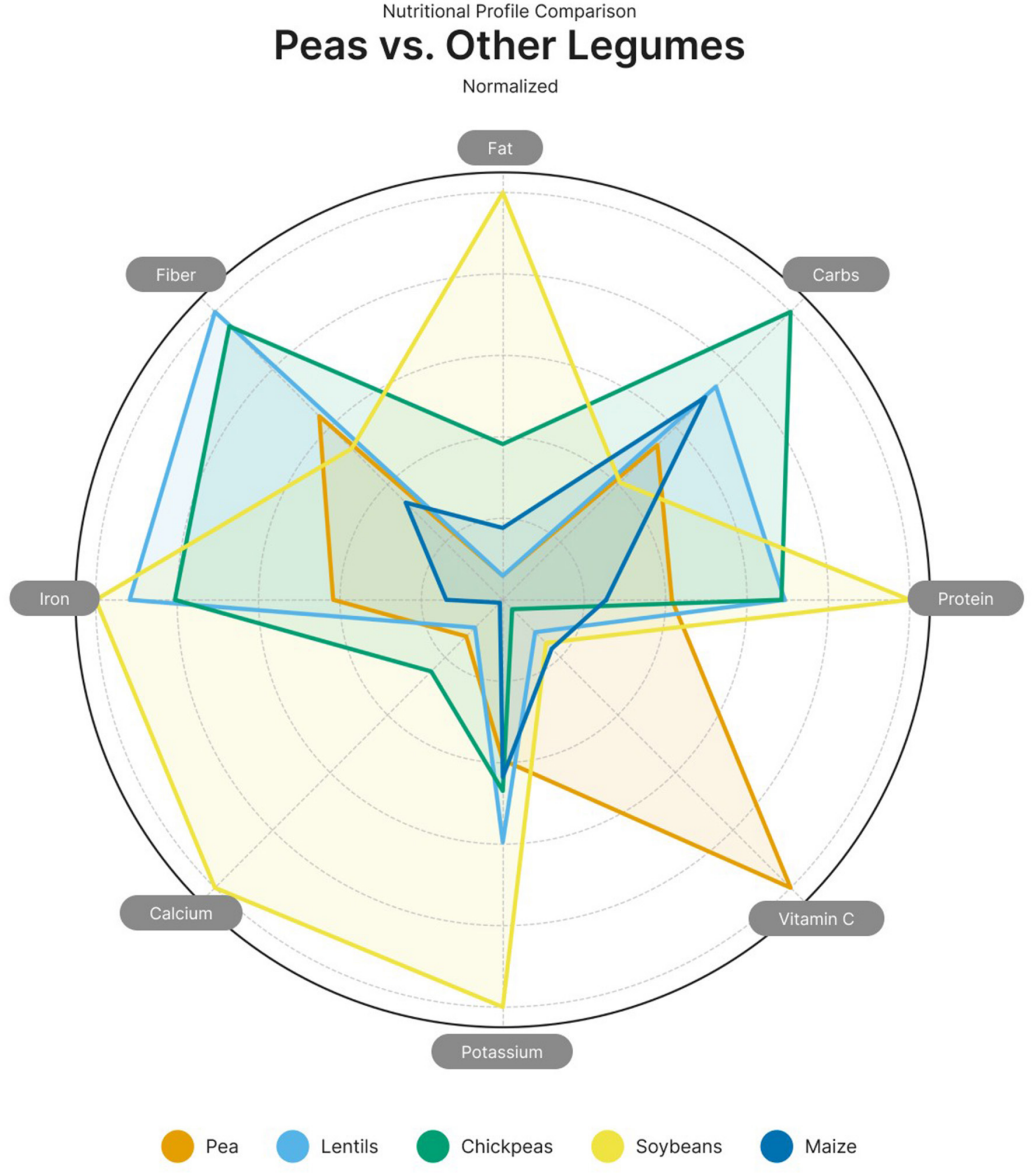
Comparison of peas' nutritional components with other legumes.

Legumes are globally recognized for their rich nutrient content and significant health benefits. Among these, peas have gained attention not only for their nutritional value but also for their unique bioactive compounds and functional properties. This section presents a comprehensive comparison of peas with other common legumes and crops, including lentils (*Lens culinaris*), chickpeas (*Cicer arietinum*), and common beans (*Phaseolus vulgaris*), with a focus on their nutritional composition and distinctive characteristics (Figures 5, 6).

### 6.1 Nutritional and non-nutritional comparison between peas and other common legumes

#### 6.1.1 Proteins

Peas contain ~20%−25% protein by dry weight, comparable to lentils and chickpeas, and slightly less than some common bean varieties (50, 68). The protein quality of peas stands out due to their high lysine content, an essential amino acid often limited in cereal grains, making them a valuable complementary protein source in plant-based diets. However, like most legumes, peas have a relatively low methionine content, necessitating dietary combinations with other protein sources to achieve a balanced intake of amino acids. Compared to soybeans, which contain a higher total protein (~36 g/100 g dry weight), pea protein is slightly lower in quantity but comparable in quality when complemented appropriately (170). Moreover, Multescu et al. (171) systematized the results of several studies and reported different protein concentrations in studied legumes, i.e., 43.86% in soy; 28.2%−33.2% in yellow, green, red, brown, and black lentils, where black lentils stood out with the highest content; up to 24% in common beans, 26%−28% and 19% in chickpea. Considering all these results, peas stand out for their high protein content. Unlike beans or lentils, pea protein is generally easier to digest, with fewer antinutritional factors such as trypsin inhibitors and tannins, particularly in yellow and green split pea varieties.

#### 6.1.2 Carbohydrates

Carbohydrates in peas mainly consist of starch, which comprises about 50%−60% of their dry weight, with a significant portion being resistant starch (37). Resistant starch resists digestion in the small intestine and acts as a prebiotic in the colon, promoting gut health by stimulating beneficial microbiota. Starch is also a primary nutrient in most legumes—a lower amount (< 20%) has been reported for soy and lupin (171). The dietary fiber content in peas is high (15%−20%), like other legumes, contributing to improved digestive health, glycemic control, and cholesterol reduction (83). Grela et al. (172) reported the highest amount of fibers in lupins (73.84–126.2 g/kg DW), with white lupin as the most abundant. Compared to this, they reported 45.71 g/kg DW in peas. On the other hand, a lower amount of fibers in pea cultivars was reported by Chen et al. (50) where the content of soluble dietary fiber was 0.71%−1.90 %, insoluble nutritional fiber 9.45–17.46 % while total dietary fiber content ranged from 11.34% to 18.79% (Figures 5, 6).

#### 6.1.3 Non-nutritional compounds

Peas are distinguished by their high levels of polyphenols, flavonoids, phenolic acids, isoflavonoids, and carotenoids, compounds with antioxidant and anti-inflammatory properties. Compared to lentils and chickpeas, peas often exhibit higher concentrations of flavonoids like kaempferol and quercetin derivatives (117, 164). Chen et al. (50) studied different pea cultivars and among 12 identified phenolic compounds, pelargonidin 3-(6^′′^-p-coumarylglucoside)-5-(6^′′^-acetylglucoside), protocatechuic acid, coumaric acid, malic acid, p-hydroxy benzoic acid, hexose gallic catechins, quercetin di-pentoside, and others were reported. These bioactives have been associated with various health benefits, including a reduced risk of chronic diseases such as cardiovascular disease, certain cancers, and type 2 diabetes. Xu et al. (173) studied 33 cool-season legumes in comparison with common beans and soybeans. In this study, colored common beans as well as black soybeans exhibited higher total phenolics and total flavonoids content than yellow and green peas and chickpeas. Antioxidant activities were strongly correlated with the amount of total phenolics.

### 6.2 Unique properties of peas

Peas stand out among legumes not only for their rich nutritional profile and bioactive compounds with health benefits, but also for their environmental value through nitrogen fixation and sustainable cultivation. The resistant starch and fiber content in peas contribute to their prebiotic potential, which supports a healthy gut microbiome. This, in turn, positively influences metabolic health by enhancing insulin sensitivity and reducing inflammation.

Unlike soybeans or peanuts, peas exhibit relatively low allergenic potential, making them suitable for consumption by individuals with legume allergies and increasing their use in hypoallergenic food formulations (68).

Pea proteins possess functional properties, such as emulsification, water retention, and gel formation, which have been exploited in the food industry to create plant-based meat alternatives, dairy substitutes, and gluten-free products. This versatility supports the growing shift toward sustainable and plant-based diets (174). Moreover, edible films based on pea protein and starch showed considerable potential for various applications in food or non-food sectors. Also, while the foaming capacity of pea commercial protein concentrate was like that of commercial protein isolate of soy, the foaming stability of pea proteins was higher (175).

Additionally, peas, being nitrogen-fixing plants, contribute to soil fertility and reduce the need for synthetic fertilizers (176). This characteristic enhances their sustainability compared to other legumes and crops, making them an environmentally friendly choice for food production. Their high fiber and bioactive content contribute to lowering blood cholesterol levels, improving glycemic control, and exerting anti-inflammatory effects. These properties suggest a role for peas in the dietary management of cardiovascular diseases, diabetes, and obesity.

### 6.3 Pea-based products and the application of pea

Peas contain valuable components and have a wide range of applications in the food, pharmaceutical, and agricultural industries due to their favorable nutritional profile and functional properties. In the food sector, peas are commonly used in the production of plant-based protein products, including meat analogs, protein-enriched snacks, and dairy alternatives such as pea milk and yogurt. Pea protein isolate is particularly valued for its emulsifying, gelling, and water-binding properties, making it a versatile ingredient in functional foods. In addition, whole peas and pea flour are used in bakery products and gluten-free formulations to improve nutritional value. Beyond food, peas are utilized in the development of nutraceuticals and dietary supplements due to their bioactive compounds, which exhibit antioxidant, anti-inflammatory, and cholesterol-lowering effects. In agriculture, peas serve as a sustainable rotational crop that enhances soil fertility, while their residues can be repurposed for animal feed or as raw material in biodegradable packaging. These diverse applications highlight the potential of peas as a multifunctional and sustainable resource. Peas are also naturally suited to European climates, making them a perfect cover crop (Table 5).

**Table 5:** Key bioactive compounds in peas and their bioactivity mechanism of action.

**Bioactive category**	**Examples in peas**	
Polyphenols and flavonoids	Quercetin, kaempferol, catechins	Antioxidant activity → reduces ROS, modulates inflammatory pathways, and improves vascular function
Carotenoids	Lutein, zeaxanthin	Antioxidant activity → eye health, modulate lipid peroxidation
Saponins	Soyasaponins	Cholesterol-lowering, modulating gut microbiota, and immune-modulating
Dietary fiber	Soluble and insoluble fibers	Modulate gut microbiota → SCFA production → metabolic regulation, improve glycemic response, cholesterol-lowering
Peptides and proteins	Bioactive peptides released during digestion	ACE inhibition → blood pressure reduction, antioxidant and anti-inflammatory effects
Vitamins and minerals	Folate, vitamin C, zinc, and iron	Cofactors for enzymatic reactions, immune modulation, and antioxidant defense
Starch, resistant starch	Slowly digestible starch	Modulates postprandial glucose, gut microbiota (SCFA), and satiety regulation

#### 6.3.1 Pea-based beverages

Pea protein is increasingly used in the formulation of plant-based beverages such as pea milk, yogurt, and probiotic drinks, offering a lactose-free, low-allergen alternative to dairy. These beverages typically exhibit favorable emulsification and foaming properties, while the addition of probiotics can further enhance their functional appeal. Innovations in fermentation and flavor masking are continually improving the sensory acceptance of these products, making them attractive to vegan and health-conscious consumers (5).

#### 6.3.2 Meat alternatives

As previously explained, pea protein concentrates and isolates are widely applied in meat analogs, including chicken-style nuggets, burger patties, and meatballs. Their neutral flavor, high protein digestibility, and good water- and fat-binding capacity contribute to desirable texture and juiciness. In combination with extrusion and binding technologies, pea-based meat alternatives can closely mimic the mouthfeel and nutritional profile of traditional animal-based products (24, 174).

#### 6.3.3 Flour-based products

Pea flour, obtained by milling whole or dehulled peas, is used to enrich baked goods and other staple products such as cake, bread, soups, and biscuits (32). Its application improves the protein and fiber content while also enhancing the glycemic control properties of these foods. However, due to its characteristic flavor and reduced gluten functionality, pea flour is often blended with other flours to balance taste and texture (167).

#### 6.3.4 Encapsulation and packaging

Beyond food formulations, pea-derived materials are finding use in the development of encapsulation systems and biodegradable packaging solutions (5). Pea protein and starch can be engineered into nano- and microparticles to protect sensitive bioactive compounds (e.g., vitamins, probiotics, polyphenols), enhancing their stability and enabling controlled release. Moreover, biodegradable films and carrier matrices derived from pea by-products represent eco-friendly alternatives to petroleum-based plastics, aligning with circular economy principles (177). Pea protein and starch-based edible films hold considerable potential for sustainable packaging solutions. Incorporation of innovative processing techniques and application of novel additives could improve the functional properties of those films, thus extending their applications beyond food packaging (175).

#### 6.3.5 Germinated pea products

The germination of peas enhances their nutritional value by increasing levels of bioavailable micronutrients and antioxidant activity. Sprouted peas and microgreens are increasingly consumed fresh, offering rich sources of vitamins C and E, phenolic compounds, and essential amino acids. These forms are particularly appealing in raw vegan diets and gourmet cuisine due to their visual, textural, and nutritional qualities (178).

### 6.4 Sustainability and peas as a functional food (environmental benefits of growing peas)

Food production significantly contributes to the overall adverse environmental impact on our planet. Therefore, populations must transition to plant-based diets, which are less detrimental to the environment, thereby reducing overall greenhouse gas emissions (GHGs) and facilitating the achievement of the Sustainable Development Goals (SDGs) within the designated timeline. The recently revised Eat-Lancet Commission report underscores the importance of healthy, sustainable, and equitable food system approaches, recognizing the food system as the leading contributor to the disruption of planetary system boundaries and the primary driver behind five of the six “breached” boundaries (179). Moreover, climate change poses a serious threat to crop production, owing to issues such as significant temperature increases that adversely influence drought conditions and decrease yields, thereby exacerbating food insecurity concerns among vulnerable populations.

Peas have traditionally been utilized within crop rotation systems, serving to improve production yields for crops such as cereals. The pea plant is considered useful in crop rotation systems because of its ability to fix atmospheric nitrogen; however, yields vary and are dependent on a number of abiotic and biotic factors. The type of soil tillage systems was also found to influence the amount of nitrogen fixation from the atmosphere, with direct sowing providing the highest yields at 50.2% (180). A recent study in Norway also highlights the importance of shifting toward plant-based diets, in a population that traditionally relies on meat and dairy products as its main protein sources. Pea production can face some challenges during production; however, the lower environmental impacts compared to animal products highlight the need for further work to improve cultivation methods and increase consumption of this crop. Lifecycle Analysis (LCA) results showed that overall, the environmental impact of peas is lower than that derived for animal proteins, although further socio-economic considerations and more precise calculations (including those concerning soil mineralization and leaching effects) are needed (181). Another LCA study carried out in Austria evaluated the environmental impact of oat: pea intercropping, as well as pea monocropping, with positive (low environmental impact) outcomes reported, and with further research recommended (182) (Table 6).

**Table 6:** Functional applications of pea-based ingredients across diverse industries.

**Sector**	**Application**	**Purpose/Function**
Food industry	Pea protein isolate (meat analogs, protein bars, plant-based dairy)	Plant-based protein source, texture, emulsification, and water-binding
	Whole peas and pea flour in the bakery and pasta	Nutritional enrichment, gluten-free formulations
Nutraceuticals	Extracts rich in flavonoids, saponins, and phenolic acids	Antioxidant, anti-inflammatory, and cholesterol-lowering effects
Pharmaceuticals	Dietary supplements based on pea bioactives	Cardioprotective and glycemic control support
Agriculture	Rotational crop	Improves soil fertility through nitrogen fixation
	Pea residues (husks, stems) as compost or green manure	Soil amendment, sustainable waste valorization
Animal feed	Pea by-products (hulls, protein-rich meal)	Protein and fiber sources in livestock and aquaculture diets
Biomaterials	Use of pea starch and fibers in biodegradable packaging materials	Renewable raw material for sustainable packaging

Among many favorable impacts on the environment, the most significant of nitrogen fixation to enhance soil fertility, peas could reduce greenhouse gas emissions (183), improve water conservation, increase biodiversity (184), and have a lower environmental footprint through plant-based protein production.

The most important of these environmental advantages is the ability of peas to fix nitrogen, which results from a symbiotic relationship with nitrogen-fixing bacteria (*Rhizobium* spp.) found in the root nodules (184, 185). Pea accumulates nitrogen in the soil (186) and has the most beneficial impact on SOC by increasing humus levels and providing organic carbon as a consequence of nitrogen fixation (187).

Pea cultivation significantly reduces the use of synthetic fertilizers (188), particularly reducing the emission of nitrous oxide (N_2_O), a potent greenhouse gas released during the production and application of nitrogen-based fertilizers (183). Excessive use of pesticides and fertilizers leads to a decrease in soil fertility and microbial biodiversity, while also causing an increase in environmental pollution and greenhouse gas emissions (189). As claimed by Everwand et al. (190), legumes can benefit biodiversity when included in cropping systems, since the over-usage of fertilizers and pesticides diminishes microbial biodiversity.

Field peas provide a cropping system that is less dependent on phosphorus use efficiency (PUE) and phosphorus fertilizer input. In addition, their nitrogen fixation activity, yield stability, and ability to maintain sufficient biomass under phosphorus-deficient conditions allow for more efficient use of phosphorus remaining in the soil by extending the time it can contribute to production (191). Moreover, rotation of legumes with other crops improves soil health and adaptability (176). Through crop rotation and intercropping with cereals, legumes play a critical role in sustainable agricultural systems. According to Foyer et al. (192), 21 Mt of nitrogen is fixed by legumes worldwide, while Stagnari et al. (193) reported a 30% increase in wheat grown with legume rotation, and specifically after rotation with field pea, nitrogen uptake of various crops increased by 23%−59%. The increase in nitrogen use efficiency in cereals through rotation with legumes provides a sustainable alternative to the need for fertilizer use (187). Additionally, integrating peas into crop rotation breaks the cycles of pests and diseases, making farming systems more resilient (193). As a plant-based protein and an important source of feed and forage for animals, about one-third of human protein needs worldwide are met by legumes (185). Peas are cheap and easily accessible food rich in carbohydrates, protein, vitamins, and minerals, making them a valuable source of nutrition for both humans and animals (4). The average protein concentration of peas is 22.3%, according to Tzitzikas et al. (30), while Hood-Niefer et al. (33) claimed it to be between 24.2% and 27.5%. Many studies have concluded that plant-based foods cause less environmental damage per kg of product than animal-based foods (181, 194). According to Nijdam et al. (194), animal-based products have up to 60 times more impact on environmental degradation than legumes. It was also found that a pea-based meal had significantly lower environmental impacts in terms of land and pesticide use than a meat-based meal (195).

These considerations collectively underscore the need to prioritize climate-smart technologies that support farmers in producing crops resilient to the increasingly harsh climate changes globally, by enhancing soil health and improving pea production yields.

### 6.5 Circular economy and waste valorization

The management of agricultural waste presents significant challenges, affecting not only the agricultural sector but also broader areas such as environmental conservation and sustainability (196). Improper disposal of these residues into the environment can lead to serious ecological consequences, including soil degradation, water contamination, and greenhouse gas emissions. Therefore, the development of effective waste management strategies is essential to mitigate these impacts and promote sustainable agricultural practices.

In this context, the valorization of pea by-products represents a promising approach to transforming materials traditionally regarded as waste into valuable resources. Pea pods and substandard peas discarded during industrial processing contain a rich array of bioactive compounds, essential nutrients, and functional components that can be utilized in the production of various value-added products (15). These components have potential applications across multiple sectors, including the food, pharmaceutical, and cosmetic industries. This approach aligns with the principles of the circular economy, which emphasize waste minimization, resource efficiency, and the creation of sustainable value chains. By converting agricultural residues into useful materials, industries can simultaneously reduce environmental burdens and enhance economic sustainability.

Empty pea pods represent a promising feed resource due to their favorable nutritional composition. They are rich in crude protein (19.8%), soluble sugars, phenolics, and macro- and micro-elements (197).

Recent studies have highlighted the potential of pea processing by-products, such as pea pod powder and pea peel protein, as functional ingredients in health-oriented foods (198–202). These findings highlight this powder as a versatile functional ingredient capable of enhancing the nutritional and physicochemical quality of diverse food products, from bakery items to emulsions, supporting both health benefits and sustainable food production.

The increasing concern for environmental sustainability and waste management has prompted the exploration of agricultural by-products as valuable resources for material applications. Lata et al. (197) reported that discarded pea peels retain notable nutritional value, containing 19.79% crude protein, 7.87% ash, 2.27% fat, and 1.84% fiber. These residues can be repurposed into biodegradable films, exhibiting desirable mechanical and physical features. Such biofilms provide an eco-friendly substitute for petroleum-based plastics, offering added socio-economic benefits through employment generation, energy recovery, and improved livelihood security.

Beyond their nutritional and polymeric applications, agricultural residues are increasingly recognized for their physicochemical properties and economic viability as adsorbent materials. Owing to their lignocellulosic composition, mainly lignin, cellulose, and hemicellulose, pea processing by-products have also been successfully converted into biochar and bioethanol (203). Furthermore, bioethanol represents a renewable alternative to fossil fuels, though reliance on edible feedstocks raises concerns regarding food-fuel competition. Upendra et al. (204) demonstrated that underutilized agro-wastes, such as field bean and green pea pods, can serve as low-cost substrates for bioethanol production. These transformations demonstrate the multifunctional nature of pea residues, establishing them as versatile feedstocks for both material innovation and bioenergy generation.

### 6.6 Market perspectives

Peas are among the most significant pulse crops in global agriculture, with annual production of ~10.5 Mt of dry peas and ~7 Mt of fresh peas (205). Their share in global pulse output is substantial, estimated at 26–40% depending on datasets and classification criteria (206, 207). Canada consistently leads global production and exports, with 3.2 million tons (Mt) recorded in 2023–2024; however, climatic variability continues to affect yields and marketable supply (208). Other major producers, Russia, France, Ukraine, and India, support global availability, though pea-specific trade series have remained fragmented in recent years. Looking forward, global agricultural production is projected to expand by ~14% by 2034, with pulse crops benefiting from productivity gains in middle-income countries (209) (Table 7).

**Table 7:** Peas' contributions to the circular economy and sustainable food systems.

**Role and function**	**Peas contribution**	**Specific metrics**	**Link to circular economy and sustainable food systems**
Nitrogen fixation and soil fertility enhancement	Peas form symbiotic relationships with nitrogen-fixing bacteria, thereby reducing synthetic nitrogen fertilizer use.	Peas have been shown to biologically fix 45–125 kg N/ha in Greek experiments. Some sources estimate legumes fix between 72–350 kg N/ha/year. A typical range for peas: 50–300 kg N/ha.	By reducing external synthetic fertilizer inputs and returning nitrogen to the system, peas support closed-loop nutrient cycles: less input, more internal recycling of N, so more sustainable and circular.
Crop rotation and agro-ecosystem resilience	Incorporating peas into crop rotations improves yields of subsequent crops and reduces fertilizer demand.	In a rotation, sowing legumes like peas before wheat can boost the subsequent wheat yield by up to ~17%. Legume-inclusion rotations reduce the need for nitrogen fertilizer by about 40 kg N/ha in the following crop. Large meta-analysis: including legumes increased the main crop's yield by up to ~20%.	Rather than a linear “monoculture + high inputs → waste” model, rotating with peas helps regenerate soil fertility, reduce external inputs, and enhance system resilience - all characteristics of circular and sustainable food systems.
Lower environmental footprint (GHG, water, land)	The lower input demand for legumes reduces emissions and resource use compared to high-input crops or animal-based protein.	Peas fix N and reduce fertilizer use, thus lowering GHG associated with fertilizer production.	By reducing resource depletion (fertilizer manufacture, synthetic N) and emissions, peas contribute to a more resource-efficient, lower-waste food system.
High nutritional value and plant-based protein	Peas are a nutritious plant-based protein source, supporting diets less reliant on resource-intensive animal protein.	High protein content and lower environmental cost.	Encouraging plant proteins like peas helps shift diets toward more sustainable food systems and supports circularity by using land/time resources more efficiently.
Valorisation of by-products and waste streams	Pea processing leaves residues (hulls, pods, etc) that can be used as feed, fiber, or other value-added uses rather than being wasted.	Legume residue contributions.	This loops value from what would otherwise be waste back into the system (feed, compost, materials), a key principle of the circular economy (closing loops, multifunctional use).
Support for local/regional food systems and reduced transport/emissions	Peas can be grown in temperate zones (including Europe) and thus support regional production and consumption, reducing reliance on long supply chains.	General statements for legumes emphasize local/regional potential.	Shorter supply chains, local production/consumption mean less transport, less food-waste risk, more system resilience: aligning with sustainable food systems and circular economy ideals.
Soil health improvement and biodiversity enhancement	Peas' root systems, nitrogen enrichment, and crop-diversity effect improve soil structure, microbial diversity, reduce erosion, and support ecosystem services.	Legume-based rotations improved soil biodiversity, fertility, and productivity (in India) - e.g., the maize-peas-groundnut system achieved high yield. Meta-analysis: legumes support biodiversity, soil health, especially when fertilizer inputs are low.	Healthy soils and diverse agro-ecosystems are foundational to sustainable food systems; the circular economy in agriculture emphasizes regeneration (not depletion) of soil and ecosystem resources, which peas help enable.

In the European Union, peas are increasingly framed as a strategically important crop for protein autonomy, plant-based dietary transitions, and climate objectives. Scenario analyses suggest strong expansion potential: pea acreage could nearly double to triple if plant-based meat substitutes capture 12.5%−40% of market share (210). Policy instruments under the Farm to Fork Strategy aim to reduce reliance on imported soy and maize, though empirical quantification of direct impacts on pea supply chains remains limited in the 2020–2025 literature (211).

Market segmentation highlights distinct demand drivers. Dry peas serve both food and feed industries, with utilization sensitive to price competition and protein content (~20%−25% crude protein, dry basis) (206). Fresh and frozen markets remain steady but are commonly aggregated within broader statistical categories. The strongest growth is observed in processed ingredients, including protein isolates, starch, and fibers, which support expanding plant-based, gluten-free, and functional food applications (Tables 8, 9).

**Table 8:** Global pea production overview (2023–2025).

**Metric**	**Value/Range (2023–2025)**	**Comment**
Global production (dry peas), Mt	≈12–15 Mt (2023); ≈ 13–16 Mt (2024–2025)	Estimated increase in 2024–2025 due to expanded land and improved yields in key growing regions.
Global production (green/fresh peas), Mt	≈~20 Mt (2023); ≈~21 Mt (2024–2025)	Slight increase expected in 2024–2025.
Global harvested area (ha)	≈ 7–7.5 million ha (2023); ≈ 7.5–8 million ha (2024–2025)	Expansion in cultivated area due to favorable market conditions.
Average yield (t/ha)	Dry peas: ~2.0–3.5 t/ha; Green peas: ~1.5–2.5 t/ha	Yield improvements expected in 2024–2025.
Top producers (dry peas)—Mt	Canada ~4.0–4.6; Russia ~2.1–2.4; China ~1.0–1.5; India ~0.7–0.9; France ~0.7–0.8; USA ~0.7–1.0	Based on FAOSTAT aggregates and Crop Trust/FAO reviews.
Regional production share (%)	North America ~35%; Europe ~20%; Asia ~25%; Other ~20%	Approximate shares of global dry pea production.
Major exporters and trade flows	Canada largest exporter; Russia & Ukraine significant; main importers: China, India	The pea trade is significant for global markets, with annual export values varying.
Trend (2010–2023)	Dry pea production +20%−35%; green pea production +15%−25%	FAOSTAT and Crop Trust reports show steady growth in production and harvested area.
Nutritional highlights (per 100 g dry weight)	Dry peas: protein 20%−25%, fiber 15%, low fat < 2%, iron 5 mg, zinc 2–3 mg; Green peas: protein ~5%, fiber ~5%, vitamin C 40 mg	Data from FAO/USDA; dry peas are an excellent plant protein source, rich in fiber and micronutrients.
Usage and applications	Human consumption (soups, snacks, plant protein), animal feed, protein concentrates	Dry peas are versatile; green peas are mainly fresh/frozen; both contribute to sustainable diets.
Key cultivation regions	Canada (Prairies), Russia (Central & Southern), China (North), India (Northern), France, USA	Major dry pea-producing regions are temperate with good soils; green peas are often grown for fresh consumption.

**Table 9:** Pea market perspectives (2020–2025).

**Period**	**Market and value**	**Key drivers**	**Key constraints**	**Regional notes**
2020	Global pea protein market valued at ≈USD 1.1 billion, projected to reach ≈USD 2.1 billion by 2025 (CAGR ≈ 13.5%).	Rising demand for plant-based proteins, allergen-free foods, and sustainable agriculture.	Limited processing capacity, taste challenges, and inconsistent supply quality.	Early investment surge in North America and Europe for plant-based food applications.
2021–2022	Global peas market ≈ USD 10.6 billion (2021), expected to reach ≈ USD 12.5 billion by 2024 (CAGR ~ 6.3%).	Expansion of vegan and flexitarian diets; integration of pea ingredients into snacks and meat analogs.	Volatile weather patterns are affecting yields, the supply chain, and transport disruptions.	Asia (China, India) is leading in production and consumption; North America is expanding ingredient processing.
2023	The pea protein market ≈ is USD 2.12 billion (2023), projected to reach USD 4.71 billion by 2030 (CAGR ≈ 12.1%).	Food and beverage sector innovation: clean-label and non-GMO trends.	Raw material cost increases; competition from soy and fava bean proteins.	North America holds ~33% share; the Pacific region is growing fastest.
2024	Asia-Pacific peas market forecast at USD 11.49 billion, with a 3.29 % CAGR toward 2030.	Rising demand in Asia for plant-based proteins; regional dietary diversification.	Infrastructure and logistics gaps; limited R&D for local pea processing.	China, India, and Australia are key players; exports from Russia and Canada remain significant.
2025 (outlook)	EU-27 field-pea production forecast ≈ 2.0 million tons (MY 2025/26); pea-protein industry projected ≈ USD 2.1 billion globally.	Supply tightening in Europe; growing functional food and feed applications.	Weather and input costs; reduced planting area (~−19 % YoY).	European Union output stabilizing; Asia and North America drive demand growth.

CAGR, compound annual growth rate; EU-27, European Union (27 member states); MY, marketing year; YOY, year-on-year; USD, United States Dollar; R&D, Research and Development; Non-GMO, non-genetically modified organism.

Innovation trajectories align strongly with circular bioeconomy strategies. Advances in wet and dry fractionation enhance protein purity and functionality while facilitating the valorization of co-streams (212). By-products such as hulls, fibers, and starch can support nutraceutical, feed, and bioplastic markets, with integrated biorefineries capable of reducing environmental impacts by ~30%−40% relative to single-product approaches (213). Overall, recent evidence positions peas as a key enabler of sustainable protein transitions and resilient agri-food systems. However, harmonized production/trade statistics, standardized market indicators, and robust assessments of policy impacts are needed to fully characterize future market trajectories and support informed investment across breeding, processing, and supply-chain infrastructure.

Key constraints include uneven regional processing infrastructure, price volatility of protein concentrates, and breeding gaps (protein yield, flavor, and stress tolerance). Strengthening local and regional processing capacity (to move up the value chain from raw peas to isolates and specialty ingredients), targeted breeding programs, and investment in quality-assurance and sustainability certification could help small and medium producers capture greater shares of the added value.

### 6.7 Current gaps in research, challenges, and future directions

Increasing the sustainability of food systems through popularizing peas and other legumes requires an interdisciplinary approach to enhance feasibility and maximize impact. Current literature primarily focuses on agricultural productivity and the nutritional composition of peas, particularly underutilized and neglected varieties (187). However, sensory and organoleptic characteristics remain mostly overlooked despite their crucial role in promoting higher intake, dietary diversity, and driving broader sustainability changes in agri-food systems through the valorization of hidden traits (193).

Flavor, texture, and other sensory attributes could be leveraged to advance sustainable development goals via market-driven approaches. Yet most heritage and lesser-known pea cultivars, preserved both *in situ* and *ex situ*, have not been systematically evaluated for these qualities, representing an underexploited reservoir with significant potential for nutrition and sustainability initiatives.

Furthermore, critical gaps remain in understanding the long-term health outcomes of pea consumption, the bioavailability of key nutrients, and the mechanisms influencing allergenicity. For example, Hara et al. demonstrated the utility of artificial neural networks (ANNs) in predicting and optimizing protein content in peas, outperforming traditional models by capturing complex interactions among environmental and soil factors (214). This approach could enable targeted agronomic interventions to enhance nutritional quality, supporting the growing demand for plant-based proteins. However, adoption of such technologies in breeding programs remains limited.

Another emerging concern is the lack of mandatory allergen labeling for pea protein in significant markets such as the EU, US, and Canada, which poses a risk of unintentional exposure, especially for individuals with legume allergies. Increased awareness among healthcare professionals and further research into legume cross-reactivity are needed to improve allergen management (215).

Moreover, legumes can contain a wide range of anti-nutritional factors, such as cyanogenic glycosides, phytic acid, oxalates, saponins, lectins, biogenic amines, proteases, and α-amylase inhibitors, which challenge their nutritional quality and health benefits. Phytic acid, trypsin inhibitors, oxalates, and lectins have been found as the main pea anti-nutritional factors (5, 216).

The rising popularity of plant-based diets has significantly increased the use of pea protein in various food products (82). Although traditionally considered a safe alternative and relatively uncommon, peas are emerging as potential food allergens, particularly in susceptible individuals sensitized to legume proteins such as vicilins and legumins. Cross-reactivity with peanut allergens has been reported, raising concerns for food labeling, especially with the growing use of pea protein in plant-based meat alternatives. Recent findings from a case series highlight the ever-increasing concern surrounding pea-related allergic reactions in children. Six pediatric cases in the United States revealed allergic responses to foods containing green peas or pea protein, with symptoms ranging from hives and angioedema to severe anaphylaxis (215). The allergens identified, originating from *Pisum sativum*, are members of the vicilin and con-vicilin protein families, known to provoke immune reactions (117). Cross-reactivity with other legumes, such as peanuts and lentils, presents additional challenges, particularly for those with pre-existing legume allergies (215). Clear allergen labeling and risk communication are crucial in clinical and regulatory contexts.

In summary, future research should address the following areas:

- Comprehensive sensory profiling of diverse pea varieties to improve consumer acceptance;
- Breeding strategies aimed at enhancing both sensory and nutritional traits, along with the valorization of by-products, to support sustainable and technologically relevant food production;
- Studies on nutrient bioavailability and long-term health impacts of pea-based diets;
- Expanded use and validation of predictive modeling (e.g., ANNs) to optimize pea quality and yield under varying conditions;
- Development of clear allergen labeling standards and deeper investigation into allergenic potential and cross-reactivity within legumes;

- By filling these gaps, the full potential of peas to contribute to sustainable, nutritious, and acceptable food systems can be realized.

## 7 Conclusion

Peas are a nutrient-dense, environmentally sustainable crop that can support global efforts toward healthier, more resilient food systems. This review emphasizes their comprehensive macronutrient profile, especially their high-quality protein and slow-digesting carbohydrates, as well as their valuable micronutrients and a wide range of bioactive compounds, including phenols, flavonoids, carotenoids, and saponins. These components contribute to antioxidant, anti-inflammatory, antidiabetic, cardioprotective, and immunomodulatory effects, offering significant potential for health care and the development of functional foods. Besides their nutritional benefits, peas also provide significant agronomic and environmental advantages. Their ability to fix atmospheric nitrogen enhances soil fertility, and their adaptability to diverse climates makes them a dependable crop amid changing ecological conditions. As a plant-based protein source, peas align well with current food trends focused on sustainability and health. However, despite their potential, peas remain underutilized in certain regions, particularly in their seed hulls and protein fractions, which are rich in phytochemicals, as well as other by-products such as pea hulls and pods. The presence of anti-nutritional factors, such as phytic acid, lectins, and trypsin inhibitors, underscores the need for processing strategies that enhance nutrient bioavailability and safety. Nonetheless, peas have appealing sensory and organoleptic qualities and hold strong cultural connotations, making them suitable for diversifying diets across food systems. Future research should aim to explore the full bioactive potential of lesser-known pea components, enhance processing methods to reduce anti-nutrients, and support breeding programs for varieties with improved nutritional and sensory qualities. With their broad range of benefits, peas could increasingly contribute to sustainable agriculture, nutrition, and public health. Their impact extends beyond individual nourishment to wider public health initiatives and food security.

## Data Availability

Data sets used for this study are available from the corresponding author upon reasonable request.
